# An econometric analysis in identifying behavioral and demographic factors associated with road crash severity in Bangladesh: Evidence from the Dhaka metropolitan city

**DOI:** 10.1371/journal.pone.0340607

**Published:** 2026-01-21

**Authors:** Nazmul Islam, Nasif Ahmed Chowdhury, Md. Ahnaf Zaman, Armana Sabiha Huq, Sk Fateh Md Rasel

**Affiliations:** 1 Department of Humanities, Bangladesh University of Engineering and Technology, Dhaka, Bangladesh; 2 Department of Urban and Regional Planning, Bangladesh University of Engineering and Technology, Dhaka, Bangladesh; 3 Accident Research Institute, Bangladesh University of Engineering and Technology, Dhaka, Bangladesh; KPC Medical College and Hospital, INDIA

## Abstract

This study explores the demographic and behavioral determinants of road traffic accident (RTA) severity in the context of the Dhaka metropolitan area, Bangladesh. Road crash data recorded by the Dhaka Metropolitan Police (DMP) were analyzed through ordered logistic regression and generalized ordered logistic regression. The results were interpreted using log odds ratios, odds ratios, predicted probabilities, and marginal effects. The findings reveal that young and middle-aged drivers exhibit significantly higher odds of severe crashes compared to underage drivers. Young-aged drivers are 14 percentage points more likely to cause fatal crashes when compared to old aged drivers in our ordered logit model. In addition, male drivers show higher odds of severe crashes than females. Factors such as overloading of vehicles, alcohol consumption while driving, and over-speeding were identified as the major contributors to increasing crash severity. Alcohol consumption had an odds ratio of 1.223 in the ordered logit model, and it had odds ratios of 2.418, 1.722, and 1.086 for the thresholds of motor collision, simple injury, and grievous injury, respectively, in the generalized ordered logit model. In contrast, the use of seatbelts, vehicle fitness maintenance, and drivers’ licensing shows mitigating effects on crash severity, with significant odds ratios < 1 in both the ordered logit and generalized ordered logit models. From the ordered logit model, we found that seat belt use, fitness certificate, and license decrease the likelihood of fatal crash by 10.7 percentage points, 8.2 percentage points, and 28.2 percentage points, respectively, whereas overspeed increases the likelihood of fatal crash by 13.5 percentage points. The results were reflected in the generalized ordered logit model, too. This research provides valuable insights for policymakers to design and implement effective policies and transport planning, including demographic driving regulations and behavioral control mechanisms to reduce road crash severity.

## Introduction

Road Traffic Accidents (RTAs) are recurrent and multifaceted incidents worldwide, causing over 1.3 million deaths and injuries to millions more, making them a major global public health as well as development challenge [1]. The road transport system serves as a critical infrastructure globally, with the majority of individuals relying on it daily, despite being cognizant of the inherent risks involved [2]. RTAs are currently the eighth leading cause of death worldwide, and might be the seventh before 2030, which makes the efficacy of road safety measures a quintessential gauge of the sophistication and development of a motorized civilization on a global scale [3,4]. RTAs and Road Traffic Injury (RTI) also increase economic and non-economic costs of health, familiar care, social acceptance, and regular treatment [5]. Globally, the age-standardized mortality rates due to road injuries decreased between 1990 and 2019, but the number of deaths and DALYs (disability-adjusted life years) increased entailed to population growth and the use of motorized transport; especially in South and East Asia exhibit some of the highest DALY burdens for road injuries [6]. Road safety is an important prerequisite for an efficient transportation system, also. Sustainable development is far away without ensuring the safety of road users, so sustainable development goals (SDGs) put emphasis on preventing road crash incidents [7]. SDG 3 and SDG 11 include road safety provisions in their targets. By 2020, SDG target 3.6 aimed to reduce fatalities and injuries caused by RTAs to half, which remains unfulfilled due to inadequate and improper interventions [8]. SDG 11.2 target sets out to ensure access to a safe urban transportation system for all [9]. Road crashes have social and economic costs. Injuries from RTAs cost the United States Dollar (USD) 1800 billion every year throughout the world, which is 3% of global GDP [10]. While the global context establishes the importance of the issue, it also underscores the need to investigate how driver-related factors contribute to crash outcomes, particularly in countries facing higher fatality burdens.

It is estimated that sixty percent of the world’s vehicles are located in middle- and lower-income countries, which account for around 92% of the world’s traffic fatalities [11]. The developing countries have some risky traits of the drivers that are responsible for the increased number of road accidents [12]. Driver behavior is often considered the most critical aspect of the road traffic safety issue, because the driver is responsible for the control of the vehicle on roads [13]. Hence, researchers around the world put emphasis on identifying driver characteristics that contribute to road crash severity. Understanding risky driving behavior and specific groups of drivers who pose a greater threat to road safety can help with targeted interventions to reduce road crashes. Hossain et al. [14] and Sagar et al. [15] reported that driver demographic factors-age and gender, socio-economic characteristics, and behavioral factors such as wrong-way driving are strong determinants of road accidents. However, behavioral factors, such as attentiveness and adherence to traffic regulations [16], as well as over-speeding tendencies and alcohol consumption while driving [17], also significantly influence road accidents. If the driver traits that influence the severity levels of road crashes can be identified, targeted interventions can be taken through intervention strategies developed through research-based policy tools. Although international evidence highlights the importance of driver behavior in shaping crash outcomes, very limited research has rigorously examined these determinants in dense and low-income megacities like Dhaka.

In Bangladesh, the daily fatalities due to road accidents are more than eight in number, with a significant contribution from driver demographics and behaviors [18]. In 2023, 7902 fatalities occurred due to road crashes in Bangladesh, causing a massive loss to the national economy. Bangladesh suffers a loss of 1.2 billion pounds every year from road crashes, equivalent to 2% of the GDP and all the foreign aid received by the country [19]. The fatality caused by road crashes in Bangladesh is 160 per 10,000 vehicles. This rate is around 1.4 in the UK and 2 in the USA, highlighting the comparatively worse condition of Bangladesh in ensuring road safety to people [20]. The alarming situation of road safety underscores the necessity of road crash severity analysis in Bangladesh. Around 22% of the crash reports of the country come from the capital city, Dhaka [21]. Drivers’ reckless behavior, overspeeding tendencies, use of unfit vehicles, and competition on roads by drivers result in more crashes in the city [22]. Despite Dhaka’s disproportionately high level of national roadway crashes and the prominent impact of driver behavior, no empirical study has systematically analyzed how driver demographic and behavioral factors influence crash severity in Dhaka using long term police reported data.

Previous studies of road crash severity in the context of Dhaka did not exclusively focus on demographic and behavioral factors related to drivers. Hossain et al. [14] studied road crash severity in Dhaka based on a questionnaire survey on victims, but their study could not capture the scenario from the entire population of drivers involved in crashes. Das et al. [23] conducted a study on the risky practices of motorcyclists in Dhaka city, but they did not consider drivers involved in crashes. Bhuiyan et al. [18] used important variables in their study related to road crash severity, but they used newspaper data from only the year 2019. Besides, their focus was not entirely on driver characteristics, and they considered rural areas along with the urban area of Dhaka, and pedestrians and passengers along with drivers. Besides, many comprehensive studies have been conducted in Dhaka concerning the crash severities of pedestrians by Akter et al. [24], Malakar et al. [25], Hossain et al. [26], Zafri and Khan [27], Zafri and Khan [28], and Zafri et al. [20]. But the focus of these studies was mostly on pedestrian behaviors, the impact of the built environment, and pedestrian-vehicular collision maneuvers. Some vehicle-specific crash studies were conducted by Rahman et al. [29] for motorcycle crashes, Ahmed et al. [30] on non-motorized vehicles, and Saha et al. [31] on unconventional modes. However, no study considered all the available vehicles to study driver faults, and these studies considered interaction of vehicle drivers with small category of road users (pedestrians, motorcyclist etc.). Besides, existing research on road crash severity in Dhaka relies on relatively small data size, which may limit a more rigorous understanding. Many crucial studies related to driver behavior were conducted on the broad context of Bangladesh [32] or outside Dhaka city [33]. Therefore, research gaps exist on the basis of the sole focus of research, the inclusion of variables related to drivers, the number of crashes investigated, and the location of the crashes. That is why we examine particularly the driver characteristics that contribute to different levels of crash severity in Dhaka city using crash reports of the Dhaka Metropolitan Police over a time period of 12 years. The main objective of this paper is to assess the driver behavioral and demographic determinants of road crash severity in Dhaka city, Bangladesh, using ordered logistic regression and generalized ordered logistic regression.

The novelty of this study is: (i) to choose a unique high-density urban area, Dhaka metropolitan city, with poor transportation facilities; (ii) to use police data for 12 years offering a comprehensive datasets available for an LMIC(low and middle-income countries) megacity, and (iii) to employ ordered logit model and generalized ordered logit model applying four different approaches that have rarely been used together in previous studies: log-odds ratios, odds ratios, predicted probabilities and marginal effects. Interpretation of the results through these four approaches offers a deeper understanding of the effects of different driver factors on crash severity. The ordered logistic regression model and generalized ordered logistic regression model have been used because road crash severity is an inherently ordered categorical variable. Unlike binary or multinomial models, generalized ordered logistic regression appropriately captures the ordinal progression of severity levels and estimates how demographic and behavioral factors influence the likelihood of heterogeneous crash severity outcomes. The findings of this paper can be helpful for road safety engineers, transport planners, urban planners, policy makers, and researchers in understanding the magnitude and type of relationship between driver demographic and behavioral characteristics and the degree of severity of an accident. These findings will be helpful to formulate regulatory and design policies for safe urban roads in Dhaka. Besides, this paper will contribute to the international research on road safety in the context of a developing country like Bangladesh.

The specific objectives of our research are:

(i) to evaluate the behavioral and demographic factors that contribute to crash severity using log-odds, odds-ratio, and probabilities of ordered logistic regression and generalized ordered logistic regression; and
(ii) to determine the marginal effects of demographic and behavioral predictors on different levels of road crash severity.

Building on the research objectives and the methodological novelty, the subsequent sections are organized as follows:’Literature Review’ section provides a comprehensive review of researches on driver demographics and behaviors associated with road crash severity;’Methodology’ section discusses the study setting and the methodology of the study, which explores the sources of data and the methods followed; ‘Results’ section provides the descriptive analysis of the core data, and interprets the regression results of ordered logit and generalized ordered logit model; ‘Discussion’ section contains the findings of this study in compliance with the results obtained; and finally, concluding remarks, recommendations, limitations and an outlook for future research are provided in ‘Conclusion’ section.

## Literature review

### Driver demographic and behavioral factors

Driver demographic factors on road crash severity have different results in the literature. The relations between drivers’ age and crash severity can vary depending on the context and location. For example, Balakrishnan et al. [34] and Bhuiyan et al. [18] found that young drivers often exhibit higher crash rates due to inexperience and risk-taking behaviors in Victoria, Australia, and Bangladesh, respectively. Similarly, Champahom et al. [35] found that younger drivers and adult motorcyclists in Thailand are in more risk of higher severity levels of crashes than older drivers. Mohanty and Samal [36] discovered in Bhubaneswar, India, that drivers in the age group 18–21 years are the riskiest group of causing a crash of higher severity, and the probability of being involved in severe crashes decreases with age. On the contrary, Alrumaidhi and Rakha [37] showed that older drivers might experience declining visual and cognitive function-related challenges, thus increasing crash risk in Virginia, USA. Ahmad et al. [38] reported that an increase in the age of drivers increases the likelihood of fatal crashes on the motorways of Pakistan. Similarly, Cespedes et al. [39] found that drivers over the age of 75 years are more likely to be involved in serious injury and fatal crashes. In Anhui Province, China, Chen et al. [40] found that middle-aged drivers had relatively higher risks of the severity of the crash. Khanpour et al. [41] and Regev et al. [42] worked on gender influence on crash severity in Iran and Great Britain, respectively, and observed that male drivers were more prone to risky behaviors than females, resulting in a higher likelihood of severe crashes. However, the effect of gender is difficult to study because in developing countries, female drivers is much lower than male drivers. Road safety studies in Qatar [43], Iran [44], and Oman [45] revealed that male drivers constitute more than 70% of drivers, so female gender naturally involve in less crashes and have lower severity of crashes. In Sri Lanka, Kodithuwakku et al. [46] found all variables related to human characteristics of crashes except gender as insignificant.

Driver behavioral factors such as over- speed, and lack of seatbelt usage are prominent contributors to crash severity, as found in Thailand [35]. Furthermore, Liu et al. [47] found that drivers who over speeded above 45 mph caused more severe accidents in North Carolina, USA, especially to pedestrians on roads. Crashes can also result from oversteering or overcorrection, often stimulated by driver age, physical condition, and reaction to sudden events [48]. Overspeeding also increases the risk of higher levels of crash severity for all classes of vehicles. In Pakistan, Junaid et al. [49] found that even the speeding of light vehicles like motorized rickshaws increases the severity level of crashes. Speeding can increase the risks of crash severities often when the congestion on roads is low, as found in Iran by Samerei and Aghabayk [50]. Seat belt/helmet use and other risky behaviors also contribute to higher levels of crash severity. The effect of seat belt negligence becomes more prominent when the driver overspeeds. Liu and Dissanayake [51] discovered that lack of seat belt usage and speeding were two of the major contributing factors of severe crashes in Kansas. Klinjun et al. [52] found that speeding, drowsiness, not using seatbelts, and overloading vehicles were associated with increased risk of severe accidents in the lower southern region of Thailand. Alcohol consumption and driving under the influence a major contributing factors to fatal and serious injury crashes. Adanu et al. [53] in Alabama, USA, and Liu & Dissanayake [51] in Kansas, USA discovered that alcohol consumption during driving and Driving Under Influence (DUI) was found to be a major cause of severe accidents, which was also found in the research by Pervaz et al. [17] in drivers of Bangladesh. Gim et al. [54] showed that alcohol use increases the risk of fatal crashes among old aged drivers in South Korea. Luan et al. [55] also reported that alcohol involvement increases the risk of higher levels of crash severity in the Dutch highway network in the Netherlands. However, Lee et al. [56] found different results showing that in Central Ohio, DUI of alcohol and drugs could drive slowly, and cause more property damage collisions rather than bodily injury accidents.

Overloading of vehicles often results in higher severity of crashes, as found in China [57,58] and Bangladesh [17]. Overloading mainly affects heavy goods or freight vehicles. Zainuddin et al. [59] found in Malaysia that overloading increases vehicle weight, which may lead to more collision forces and higher crash severity. Overloading also occurs because of carrying excessive passengers on vehicles. Lam [60] found that carrying too many passengers increased crash severity in New South Wales of Australia. Distracted driving is a major cause of crash severity in different parts of the world. Mansoor et al. [61] reported that distracted driving increases the severity of motorcycle crashes in Rawalpindi city of Pakistan. Besides, driving under fatigue and distressed conditions also increases the severity of crashes, as found in the motorways of Great Britain [62]. Diverse cultural backgrounds influence norms and practices, which can cause varying driving behavior and crash rates. For instance, the United Arab Emirates (UAE) experiences a high rate of roadway crashes, with one of the influencing factors being its multicultural population with different driving behaviors [63]. Hostile driving behavior, like aggressive driving and risky driving style, increases the risk of crashes [44]. These types of behavior are less found among trained and licensed drivers. Driver licensing ensures that drivers drive peacefully without harm to their surroundings or other humans. Martín-delosReyes et al. [64] found that drivers without a license or with a suspended license are at higher risk of crash involvement than drivers with a valid license. When drivers drive vehicles without fitness, these effects may combinedly cause more severity. Pervaz et al [17] found that drivers driving vehicles without a valid fitness certificate are involved in higher crash severities in Bangladesh.

#### Methodological approaches.

Multiple studies with varying methodological formulations intricately outline the underlying factors of road crash severity. Most of the studies were conducted based on secondary datasets compiled by law enforcement agencies. Traditional severity modelling used logistic regressions. Lee et al. [56] conducted binary logistic regression analysis based on crash data obtained from the Ohio Department of Public Safety. Ma et al. [65] collected crash data from the Federal Railroad Administration (FRA) database of the USA, and used a mixed logit modelling approach to identify the factors affecting the severity of crashes. Some monitoring bodies other than the police also report and provide road crashes for use by researchers. Klinjun et al. [52] collected data from the Office of Disease Prevention and Control 12 (ODPC12) of Songkhla Province of Thailand and used the Haddon Matrix to identify factors behind road traffic injury severity. Among all the types of logistic regression models, ordered logistic regression or the ordered logit model has been used extensively to identify causes behind different levels of crash severity. Balakrishnan et al. [34] collected crash data from VicRoads, compiled from all police reported crash incidents in Victoria, Australia, and used a random parameter ordered logit model. Chen et al. [40] used geographically and temporally weighted ordered logistic regression (GTWOLR), and the data were collected from Anhui traffic police. Luan et al. [55] used a two-step cluster analysis and a latent class ordered regression model with covariates based on the Dutch crash registration database in the Netherlands. Gim et al. [54] focused on the crash severity of older-aged drivers, and they conducted generalized ordered logistic regression to identify the determinants of crash severity of older drivers in South Korea by using the data of the Korean National Police Agency. Rifaat and Chin [66] collected data on crashes in Singapore from 1992 to 2001 and conducted an ordered probit model analysis. Çelik and Oktay [67] used ordered logit, generalized ordered logit, partial constrained generalized ordered logit, and heterogeneous choice model as ordered response models, and multinomial logit and mixed logit model as unordered response models to identify risk factors contributing to crash severity in north-eastern Turkey based on the police reports.

Advanced statistical models other than traditional regression analysis are being utilized at present to model crash severity. A popular state-of-the-art approach is joint modelling of the marginal distribution of econometric models. Pervaz et al. [17] used copula copula-based joint model to identify the factors affecting road crash severity and crash types in Dhaka based on the database stored in the Accident Research Institute (ARI) of Bangladesh, which was collected from the Dhaka Metropolitan Police. Tamakloe et al. [68] used a copula approach to jointly model the number of vehicles and crash severity in crashes involving express buses in Korea, and they used the data of Koren Expressway Corporation. Guilhermina et al. [69] collected data from the Portuguese Police Republican National Guard and applied classification and regression trees (CART) and found two-vehicle collision and vehicle weight to be evident factors in road crash severity in the Porto Metropolitan Area, Portugal. Researchers also used data from hospitals for modelling crash severity. Daddah et al. [70] conducted a nested case-control study based on hospital cohorts of the victims affected by road crashes. Odinfono et al. [71] used Geographic Information System (GIS) based Geographically Weighted Regression for analyzing crash severity using real-time road crash data of the Federal Road Safety Corps of Southwest Nigeria.

At present, the application of machine learning and artificial intelligence in severity modelling is being done by a large number of researchers. Sum et al. [72] used data from Thailand’s Highway Accident Information Management System and conducted random forest model analysis combined with Shapley Additive explanation (SHAP). Kumar et al. [73] used multinomial logit, decision tree, and random forest models to analyze crash severity in three selected expressways of India. Barman and Bandyopadhyaya [74] used Back Propagation-Artificial Neural Network (BP-ANN) to model the severity of crashes in low-speed urban roads of Patna, India. Se et al. [75] studied factors of motorcycle crash severity in Thailand using random forest, support vector machine, deep neural network, recurrent neural network, long short-term memory, and extreme gradient boosting. Astarita et al. [76] collected a crash database from Automobile Club Italia- ISTAT and developed an artificial neural network (ANN) and Grey Wolf optimization algorithm (GWO)–ANN to study the causes of road crash severity in Calabria of Italy. Samerei and Aghabayk [50] used latent class clustering and an interpretable categorical boosting method (CatBoost), combined with SHAP, to study the risk factors of run-off-road crash severity in Iran.

## Materials and methods

### Study area and time frame

We use the Dhaka Metropolitan Area as a study location for crash severity analysis. Dhaka is the capital and primate city of Bangladesh, having the highest urban and transportation services of the country [87]. But these services are not adequate for ensuring an efficient transportation system, and the occurrence of road crashes is very high in this city. It is estimated that the crash fatality in Bangladesh is around 25 times higher than in developed countries, and this fatality is higher in Dhaka due to poor traffic infrastructure and road design [88].

For this study, a time period of 12 years was selected for rigorous analysis of the demographic and behavioral factors of road crash severity. This study used accident data from 2011 to 2022 for analysis. We aimed to use a large sample of data for a more robust analysis of the behavioral and demographic determinants of road crash severity in Dhaka, so we used data from 12 years. This study time was selected because the latest crash data available at the time of conducting this research was up to 2022, and the crash data before 2011 lacked sufficient data due to technical limitations at that time. That is why we selected the study time from 2011 to 2022.

### Data collection and pre-processing

The local police authority of Bangladesh collects accident-related data after an investigation of a road crash and preserves it in a form known as the Accident Report Form (ARF). The Accident Research Institute (ARI) of the Bangladesh University of Engineering and Technology (BUET) collects the filled-out ARF regularly [89]. The information from the form is stored in the Microcomputer Accident Analysis Package (MAAP) version 5 software [29]. The accident records of the years 2011-2022 stored in MAAP 5 were collected from the ARI of BUET. The data provided was in DAT format, and separate data was available for each year. The DAT file for accident records of each year was converted and merged into a compiled Microsoft Excel 2024 Spreadsheet. There were 4702 driver involvements in the accident records from the years 2011 to 2022. Out of them, 390 observations had any missing data or entries. These data were discarded, and the remaining 4312 driver crash records were used for this study. The number of drivers’ crash records for each year according to different levels of crash severity is summarized in Table 1. Our study covered the years of lockdown during the COVID-19 pandemic in 2020. Due to the lockdown, there was less road traffic, which resulted in fewer crash records. However, since we have used the dataset for a large number of years, we had an adequate sample size. Besides, our objective is to relate the crash severity with driver behavioral and demographic characteristics, so having fewer crash records in the lockdown years did not impact our analysis.

**Table 1:** Yearly number and percentages of driver crash records of different levels of severity.

Year	Total Number of Driver Crash Records	Number of Crashes of Different Levels of Severity (Yearly Percentage)			
		Motor Collision	Simple Injury	Grievous Injury	Fatal Crash
2011	74	12 (16.22)	10 (13.51)	16 (21.62)	36 (48.65)
2012	493	49 (9.94)	29 (5.88)	76 (15.42)	339 (68.76)
2013	450	31 (6.89)	28 (6.22)	84 (18.67)	307 (68.22)
2014	436	37 (8.49)	40 (9.17)	72 (16.51)	287 (65.83)
2015	488	25 (5.12)	34 (6.97)	85 (17.42)	344 (70.49)
2016	406	27 (6.65)	14 (3.45)	87 (21.43)	278 (68.47)
2017	344	16 (4.65)	21 (6.1)	76 (22.09)	231 (67.15)
2018	96	4 (4.17)	3 (3.13)	15 (15.63)	74 (77.08)
2019	431	10 (2.32)	14 (3.25)	110 (25.52)	297 (68.91)
2020	125	8 (6.4)	7 (5.6)	33 (26.4)	77 (61.6)
2021	449	14 (3.12)	19 (4.23)	123 (27.39)	293 (65.26)
2022	520	24 (4.62)	21 (4.04)	126 (24.23)	349 (67.12)

Note: Values in Parentheses Show Yearly Percentages of Crashes of Different Levels of Severity.

The dataset was prepared, and preliminary pre-processing was done in Microsoft Excel 2024. All the symbolic values were decoded to their original meanings by following the coding of ARF. The categorical data were converted to binary categories through the One Hot Encoding Method. Driver Age was a numeric variable in a ratio scale. For better interpretation, the age of drivers was categorized. Following the procedure of Bhuiyan et al. [18], the following categorization of drivers’ age was done: Underaged (Age<18), Young Adult (18<=Age<=35), Middle Aged Adult (35<Age<=55 years), Older Adult (Age>55 years). Since there were a total of 18 categories of vehicle type, the vehicle types were reclassified into 6 categories by improving the three-level vehicle classification used by Rahman et al. [29]. These are: (i) Non-Motorized Vehicles (NMV): Bicycle Rickshaw/Van, Cart, Animal Driven vehicles; (ii) CNG/Tempo: Baby Taxi/CNG, Tempo, Others (Leguna, Auto-rickshaw, Nosimon, Korimon); (iii) Car/Microbus: Car, Microbus, Jeep, Tractor; (iv) Motorcycle; (v) Medium Weight Vehicle: Minibus, Pickup, Small Truck; (vi) Heavy Weight Vehicle: Bus, Heavy Truck, Articulated Truck, Tanker. Other variables from the dataset were kept unaltered.

### Variable selection

From the literature review, many demographic and behavioral factors that influence road crash severity were identified and summarized in Table 2. But from the data collected, the available factors for analysis were driver age, gender, seat belt (or helmet for motorcycle drivers), alcohol, overspeed, overload, license, and fitness certificate. Some other variables were present in the ARF that were not related to driver behavior or demographics. These were used as control variables, which were time of day (based on light availability), intersection type, vehicle type, collision type, and traffic control measure. All the variables used were categorical in nature. The dependent variable in our study is the crash severity. In Bangladesh, police investigate any crash occurrence and mark it in any of the following four categories: motor collision (property damage only), simple injury crash (minor injury), grievous injury crash, and fatal crash. The order of severity is fatal crash> grievous injury crash> simple injury crash> motor collision. A fatal crash is an accident where the victim faces death at the site or within 30 days of the accident due to injury [90]. Grievous injury crash is defined by the Penal Code 1860 of Bangladesh as any accident which causes any of the following eight injuries: emasculation, permanent privation of any eyesight, permanent privation of any ear capacity, privation of any kind of body member or joint, destruction or permanent impairing of the powers of anybody member or joint, permanent disfiguration of head or face, fracture or dislocation of bone or tooth, and any injury that endangers life or causes the victim to suffer severe body pain or unable to involve in ordinary pursuits for 20 days [91]. A simple injury crash is any injury that does not fall in the eight categories of grievous injury crashes. Simple injuries include cuts, bruises, abrasions, minor sprains, soft tissue injury without fracture, and minor head injuries without loss of consciousness. These injuries require only primary first aid, heal within a few days, and allow the victim to return to normal activities within 20 days. Siddique [90] stated that a person suffering a simple injury may need to take medical leave of 3 days. Motor collision is an accident where only vehicles or property are damaged without any fatality or injury of any person.

**Table 2:** Overview of the literature review on demographic and behavioral determinants of crash severity.

Determinants	Author	Findings
Age	[38]	The risk of being involved in higher levels of motorway crashes increases with the age of the driver.
	[56]	The influence of driver age on accident severity follows a polynomial curve. The probability of bodily injury or fatal crashes increases at age 15, then declines up to age 33, and further increases at age 47.5 years.
	[40,63]	Middle-aged drivers account for more severe accidents than young drivers
	[35,36,77]	Young aged drivers practice risky driving and are involved in higher severities of road crashes.
	[37,39]	Old-aged drivers are involved in higher levels of crash severity due to impairment in driving skills.
	[78]	Young drivers (under 21 years) and senior drivers (over 65 years) are associated with higher crash severity than other age groups.
Gender	[18,[39,41,79]	Male drivers are more likely to be involved in higher-severity crashes than female drivers.
	[46]	Driver gender is not a significant contributor to road crash severity.
Seat Belt/ Helmet Use	[18,80]	Drivers who do not wear seat belts were involved in severe RTAs.
	[52]	Not using seat belts, having malfunctioning seat belts, or the presence of inappropriate child-adjustable seat belts elevate the severity of a road crash.
	[81]	Use of helmets decreases crash severity, with an 88% reduction in serious head crashes
	[82]	The use of a helmet reduces the chances of fatal and severe injuries in motorcycle collisions.
Alcohol Consumption	[17,51]	Driving under the influence of drugs and alcohol affects crash type and causes severe RTAs.
	[56]	Intoxicated drivers often drive more slowly, so alcohol and drug-related crashes result in more Property Damage Only (PDO) than bodily injury crashes
	[54]	Alcohol consumption increases the risk of higher severity of crashes among older drivers.
Overspeed	[65]	Driving over 50 mph increases the probability of the highest severity (fatal) of RTA by 14.8% for aggressive drivers and 8.4% for non-aggressive drivers.
	[49,53,78]	Higher vehicular speed is associated with higher severity of RTA-induced injury.
	[50]	Overspeeding increases the risk of higher levels of severity of a crash when traffic on roads is low.
	[83]	Driving on roads with high-speed limit triggers aggressive behavior of drivers, which increases severity of crash.
Overloading	[57,58,84]	Overloading of vehicle increases the severity level of road crashes
	[52]	Overloading and vehicle modification can cause a pre-crash before a severe RTA.
	[59]	Overloading increases vehicle weight which results in more force of collision and results in severe crashes.
	[60]	Carrying too much passengers than capacity of vehicle causes overloading and increases crash severity.
Licensing	[18]	Severe crashes are found frequently in non-professional (no license) drivers than in professional (having a license) drivers.
	[53,64]	Unlicensed drivers have a higher probability of being involved in severe accidents than licensed drivers.
Fitness of Vehicle	[17]	Drivers who drive vehicles without fitness tend to cause more severe RTAs.
Income	[14]	Higher-income households have a higher risk of direct involvement in severe RTAs.
	[15]	Drivers residing in areas characterized by lower socioeconomic conditions exhibit a heightened probability of RTA severity.
	[53]	Drivers with no employment (less income) have 1.32 times more odds of a higher severity of RTA than employed or self-employed drivers.
	[85]	Low income of drivers causes them to take more workloads and engage in risky driving, which increases severity of crash.
Education/ Awareness	[34]	Areas that have a higher proportion of university graduates are less likely to experience severe crashes.
Distracted Driving	[61]	Distracted driving increases the chances of severity of crashes.
	[86]	Using a mobile phone, eating/drinking while driving increases the severity of an accident.
Fatigue and Drowsiness of Drivers	[52]	Fatigue and drowsiness of drivers due to long-term driving increases the severity of RTAs.
	[85]	Fatigue and sleep problems of drivers lead to impairment of driving skills, lack of concentration, decline in memory and alertness, which increases crash severity

Harrison and Pius [92] and Agresti [93] suggested a chi-square test of independence to evaluate the association between categorical variables. Our dependent variable, road crash severity, is categorical, and all of our independent variables are also categorical variables. For this purpose, we conducted a Chi-square test of independence. Chi-square test of independence finds out the level of association between crash severity and each of the driver demographic and behavioral factors, and control variables.

Although a p-value of less than 0.05 indicates significance, a p-value less than 0.25 indicates inclusion in statistical modelling, as indicated by Zafri et al. [94]. In a similar approach, we interpret the variables with Chi square p-value less than 0.05 of the Chi square test as statistically significant at 5% level and consider the variables with a p-value less than 0.25 as variables to be considered for inclusion in the generalized ordered logistic regression.

### Ordered logistic regression

#### Model formulation.

If there is a specific order in the response variable, ordered logistic regression has been used in the literature for multivariate statistical modelling. Liu et al. [47] suggested ordered logistic regression when the response variable is accident severity, having levels of injury or fatality. Considering the presence of order in the response variable accident severity of our research (Motor Collision<Simple Injury Accident<Grievous Injury Crash<Fatal Injury Crash), ordered logistic regression was conducted in this study. It is appropriate when the dependent variable has an order among its categories. Ordered logistic regression, or the ordered logit model, was developed in licensed Stata 18 software.

In our case, the dependent variable is the road crash severity, and the independent variables are driver demographic and behavioral factors. In the standard ordered logit model, the response variable of accident severity is discrete in nature. The severity levels of the accident (y_i_) are assumed to have an association with a continuous latent variable (y_i_^*^) [95]. The values of this latent variable are expressed through a group of ordered categories. The latent variable can be expressed through a linear equation like Yasmin et al. [96]:


$$y_{i}=X_{i}\beta_{i}+\varepsilon_{i}\;\text{for}\;\text{i}=\text{1},\;\text{2},\;\ldots \text{n}$$ (1)

Here, i represents the record of a specific driver, X_i_ represents a vector of exogenous variables without a constant, *β* is a vector of unknown parameters, and ε is the standard logistic random error term. So, for a particular observation of a driver, we will initially get a latent dependent variable expressing the crash severity. If j = 1, 2,... indicates the accident severity level and τ*_j_* indicates the thresholds associated with j, the unknown τ*_j_* are assumed to divide the propensity into J-1 intervals; then the latent variable y_i_^*^ can be related to the ordinal variable yi through the following response mechanism:


$$y_{i}\;=j,\;\text{when}\;\tau_{j-\text{1}}<y_{i}*<\tau_{\text{j}};\;\text{j}=\text{1},\;\text{2},\ldots$$ (2)

The thresholds are generally assumed to have ascending order, that is, τ_0_ <τ_1_ <…<τ_*j*_. In our case, the crash severity has 4 levels: motor collision, simple injury, grievous injury and fatal collision. Therefore, we will have 3 thresholds. One threshold or intercept will cut off motor collision and simple injury level, another threshold will cut off simple injury and grievous injury level, and the final threshold will cut off grievous injury and fatal collision severities. The probability expressions for individual i and alternative j are:


$$\pi_{ij}=Pr(y_{i}=j|X_{i})=\Lambda (\tau_{j}-X_{i}\beta )-\Lambda (\tau_{j-\text{1}}-X_{i}\beta )$$ (3)

Here, Λ(.) is the standard logistic cumulative distribution function.

Ordered logistic regression uses the maximum likelihood method for estimation. The cumulative probability that Yi falls in category j and below can be expressed as:


$$\text{Pr}({\text{Y}}_{\text{i}\leq \text{j}})=\text{Pr}(\beta_{\text{1}}X_{\text{1}i}+\beta_{\text{2}}X_{\text{2}i}+\ldots +\beta_{k}X_{ki}+\epsilon_{i}\;\leq \tau_{j})$$ (4)

The maximum likelihood function finds the probability of observing specific levels of dependent variables from the independent variables.

#### Estimating log-odds and odds ratios.

The effect of a regressor on the ordered dependent variable is non-linear in ordered logistic regression. So, the interpretation of the ordered logit model becomes difficult. The odds ratio can be used for easier interpretation of the ordered logit model, as defined by:


$$\frac{\text{Pr}[Y_{i}\leq \text{j}|\text{X}}{\text{Pr}[Y_{i}>\text{j}|\text{X}]}=\frac{\text{Pr}[Y_{i}\leq \text{j}|\text{X}]}{\left[\text{1}-\text{Pr}(Y_{i}\leq \text{j}|\text{X})\right]}$$ (5)

Here,


$$Pr[Y_{\text{i}\leq \text{j}}|X)=\sum_{m=\text{1}}^{j}Pr[Y_{i}=m|X]$$ (6)

Equation (6) denotes the cumulative probability that the outcome is less than or equal to j.

Taking the log of the odds ratio and simplifying, the logit or log-odds ratio can be found:


$$\text{logit}\;[\text{Pr}(Y_{i}\leq j)]=ln\frac{\text{Pr}\left(Y_{i}\leq j\right)}{\text{Pr}\left(Y_{i}>j\right)}=ln\frac{\text{Pr}\left(Y_{i}\leq j\right)}{\left[\text{1}-\text{Pr}\left(Y_{i}\leq \text{j}|\text{X}\right)\right]}=a_{j}-\sum_{n=\text{1}}^{k}\beta_{n}X_{in}\;\;\;\text{j}=\text{1},\text{2},\ldots (\text{J}-\text{1})$$ (7)

Log odds allow understanding the direction of the relationship between predictors and severity, whereas the odds ratio is the simple exponentiation of logit coefficients (exp(logit coefficient)). If the Odds ratio of the predictor is greater than 1, the odds of Y increase, and when the Odds ratio is less than 1, the odds of Y decrease [97].

#### Estimating predicted probabilities.

In this stage, we will predict the probabilities of the outcomes found in the ordered logit model. Setting all predictors to mean values, the probability of the accident severity for different values of accident severity (motor collision, simple injury crash, grievous injury crash, fatal crash) was predicted. The ordered logit prediction is the probability that *Y*_*i*_ + ε_*i*_ lies between a pair of cut points, τ*i*−1 and τ*i*. The expressions are:


$$\text{Pr}(Y_{i}+\epsilon_{i}<\tau )=\frac{\text{1}}{\text{1}+exp(Y_{i}-\tau )}$$ (8)

$$\text{Pr}(Y_{i}+\epsilon_{i}>\tau )=\text{1}-\frac{\text{1}}{\text{1}+exp(Y_{i}-\tau )}$$ (9)

$$\text{Pr}(\tau_{\text{1}}<Y_{i}+\epsilon_{i}<\tau_{\text{2}})=\frac{\text{1}}{\text{1}+exp(Y_{i}-\tau_{\text{2}})}-\frac{\text{1}}{\text{1}+exp(Y_{i}-\tau_{\text{1}})}$$ (10)

The predicted probabilities show the probability of the occurrence of fatal crash, simple injury crash, grievous injury crash, and fatal crash from the observed data.

#### Marginal effects calculation.

Marginal effects show the change in probability from lower levels of accident severity to higher levels of accident severity when there is a unit change in the predictor variables [47]. Through the partial derivative of equation (1), the marginal effect of the *j*th regressor on *Y*_*i*_ can be expressed as:


$$\frac{\partial Y_{i}}{\partial X_{ij}}=\beta_{j}$$ (11)

This indicates that, holding all other variables constant, a one-unit change in X_ij_ is expected to cause a change in $Y_{i}$ by $\beta_{j}$ units. The marginal effects of individual crash factors on each of the levels of crash severity were analyzed in Stata 18. From the marginal effects, we can find how much more or less motor collision, simple injury collision, grievous injury collision, and fatal crashes are likely to change when there is a 1 unit change in the values of the independent variables.

### Generalized ordered logistic regression

#### Model formulation.

A big limitation in using the traditional ordered logit model is that it cannot explain the heterogeneity across different levels of the outcome variable [54]. For examining the heterogeneous results across different severity levels, we additionally used a generalized ordered logit model. The generalized ordered logit model for an ordinal dependent variable with M categories can be written as shown by Williams [98]:


$$P\left(Y_{i}<j\right)=\frac{\text{exp}(\alpha_{j}+X_{i}\beta_{j})}{\text{1}+[\text{exp}\left(\alpha_{j}+X_{i}\beta_{j}\right)]},\;\text{j}=\text{1},\;\text{2},\;\ldots ,\;\text{J}-\text{1}$$ (12)

Here, P is the cumulative probability function of the generalized ordered logit model, j is the number of categories of the dependent variable, α_j_ is the cutpoint (threshold) for category j, β_j_ is the vector of the coefficients of the regression for the j-th individual, and J is the total number of categories in the ordered dependent variable. The cumulative probability function shows the probability that the observed outcome of the dependent variable for individual i, is in a category lower than the j-th category. For our dependent variable, we have 4 categories: motor collision, simple injury, grievous injury, and fatal crash, so we have j=4. According to the cumulative probability function of the generalized ordered logit model, we will have 3 cutpoints.

In a generalized ordered logit model, the thresholds vary across individuals because of are a function of explanatory variables. Thus, heterogeneity is found in the results across different groups of dependent variables. The linear function of threshold can be expressed through equation (13) as shown by Aaditiya and Rahul [99].


$$\tau_{i,j}=\alpha_{j}+\delta_{j}Z_{i}+\delta_{j}^{\prime }X^{*}$$ (13)

Here, τ_i,j_ is the threshold of category j of the dependent variable for individual I,α_j_ is the set of cutoffs, δ_j_ represents the parameters for the independent variable Z_i_ and δ’_j_ represents parameters for a latent variable X^*^.

### Estimating log-odds and odds ratio

The log odds and odds ratio are found from the logistic equation of the ordered logit model. The logit equation for the generalized ordered logit model can be obtained as shown in equation (14), following Navarrete-Parra [100].


$$logit(P\left(Y\leq j|x\right))=\text{ln}(\frac{P\left(y\leq j|x\right)}{P\left(y>j|x\right)})=\alpha_{j}+(\beta_{\text{1}}X_{\text{1}}+\beta_{\text{2}}X_{\text{2}}+\ldots +\beta_{k}X_{k})$$ (14)

Here, the regression coefficient of equation (14) indicates the log odds ratio for k independent variables. That is, log odds = β_jk_, that is, the coefficient of a particular variable, k, under a specific category of dependent variable, j. The odds ratio is the exponentiation of the log odds ratio. That is, odds ratio exp (log odds ratio) = exp(β_jk_). In our model, we will get log-odds and odds ratios for every independent variable for cumulative probabilities of motor collision, simple injury, and grievous injury, due to 3 cutoff points.

We used licensed Stata 18 software for generalized ordered logit model formulation using the function gologit2. Some changes are used in the gologit2 function in case of defining cumulative odds, which changes the interpretation of log odds and odds ratio. The equation for the cumulative probability of the generalized ordered logit model used in the ‘*gologit2*’ function of Stata is as defined by Williams [101]:


$$P\left(Y_{i}>j\right)=\frac{\text{exp}(\alpha_{j}+X_{i}\beta_{j})}{\text{1}+[\text{exp}\left(\alpha_{j}+X_{i}\beta_{j}\right)]},\;\text{j}=\text{1},\text{2},\ldots ,\text{J}-\text{1}$$ (15)

This equation differs from equation (12) because it shows the cumulative probability of being in a higher category than category j. Therefore, the corresponding logit equation becomes:


$$logit(P\left(Y>j|x\right))=\text{ln}(\frac{P\left(y>j|x\right)}{P\left(y\leq j|x\right)})=\alpha_{j}+(\beta_{\text{1}}X_{\text{1}}+\beta_{\text{2}}X_{\text{2}}+\ldots +\beta_{k}X_{k})$$ (16)

Thus in our model, the odds ratio would indicate the odds of being in all higher categories of road crash than category j. In case of motor collision (j = 1), the odds ratio would indicate the odds of being in a crash of higher severity(j > 1) than motor collision, which is simple injury, grievous injury and fatal crash. In case of simple injury(j = 2), the odds ratio would indicate the odds of being in a higher category (j > 2) of road crash than simple injury, which is grievous injury and fatal collision. In case of grievous injury (j = 3), the odds ratio would indicate the odds of being in a higher category (j > 3) of road crash than grievous injury, which is fatal crash.

#### Predicted probabilities and marginal effects.

In this stage, we will predict the probabilities of the outcomes found in the generalized ordered logit model. Setting all predictors to mean values, the probability of the accident severity for different values of accident severity (motor collision, simple injury crash, grievous injury crash, fatal crash) was predicted. The expressions, shown by Long and Freese [102], are:


$$\text{Pr}(\text{y}=\text{1}|\text{x})=\frac{\text{exp}(\tau_{\text{1}}-x\beta_{\text{1}})}{\text{1}+\text{exp}(\tau_{\text{1}}-x\beta_{\text{1}})}$$ (17)

$$\text{Pr}\left(\text{y}=\text{j}|\text{x}\right)=\;\frac{\text{exp}\left(\tau_{j}-x\beta_{j}\right)}{\text{1}+\text{exp}\left(\tau_{j}-x\beta_{j}\right)}-\frac{\text{exp}\left(\tau_{j-\text{1}}-x\beta_{j-\text{1}}\right)}{\text{1}+\text{exp}\left(\tau_{j-\text{1}}-x\beta_{j-\text{1}}\right)}\;\text{for}\;\text{j}=\text{2}\;\text{to}\;\text{J}-\text{1}$$ (18)

$$\text{Pr}(\text{y}=\text{J}|\text{x})=\text{1}-\frac{\text{exp}(\tau_{j-\text{1}}-x\beta_{J-\text{1}})}{\text{1}+\text{exp}(\tau_{J-\text{1}}-x\beta_{J-\text{1}})}$$ (19)

The equations above are based on the condition that $\left(\tau_{j}-x\beta_{j})\geq (\tau_{j-\text{1}}-x\beta_{j-\text{1}}\right)$. It ensures that Pr(y = j|x) is between 0 and 1.

We calculated the marginal effects after the generalized ordered logit model in the same process as for the ordered logit model.

## Results

### Descriptive analysis

Table 3 shows the descriptive analysis of the data used in this study. The percentage of accidents based on the degree of crash severity for different categories of each variable is given in parentheses. The number of drivers who were found to be drinking alcohol was less than the drivers who were not consuming alcohol. This is because the amount of alcohol consumption is naturally less in Bangladesh due to the societal context and religious customs. Among the drivers driving under the influence of alcohol, the majority were found to be involved in fatal crashes (73.73%). However, drivers driving without the influence of alcohol showed a relatively lower percentage of involvement in severe crashes (66.11%). This indicates that although alcohol consuming drivers are relatively less in number, they contribute more to crashes of higher levels of severity. The values of chi-square and p-value show that alcohol consumption has a strong and significant impact on crash severity.

**Table 3:** Descriptive statistics and association of predictors with road crash severity.

Variable		Accident Frequency				*X*^*2*^ *Value*
		Motor Collision	Simple Injury Crash	Grievous Injury Crash	Fatal Collision	
Behavioral Factors	**Alcohol:**					
	Yes	26 (3.22)	36 (4.46)	150 (18.59)	595 (73.73)	22.94***
	No	231 (6.59)	204 (5.82)	753 (21.48)	2317 (66.11)	
	**Seat Belt/Helmet:**					
	Yes	58 (10.62)	50 (9.16)	169 (30.95)	269 (49.27)	98.09***
	No	199 (5.28)	190 (5.05)	734 (19.49)	2643(70.18)	
	**License:**					
	Yes	138(21.56)	111(17.34)	102(15.94)	289 (45.16)	558.98***
	No	119 (3.24)	129 (3.51)	801 (21.81)	2623(71.43)	
	**Overloading:**					
	Yes	10 (3.4)	8 (2.72)	32 (10.88)	244(82.99)	34.46***
	No	247 (6.15)	232 (5.77)	871 (21.68)	2668(66.4)	
	**Fitness Certificate:**					
	Yes	123(10.75)	93 (8.13)	218 (19.06)	710(62.06)	87.91***
	No	134 (4.23)	147 (4.64)	685 (21.62)	2202(69.51)	
	**Overspeed:**					
	Yes	160 (4.28)	215 (5.75)	776(20.75)	2589(69.22)	147.183***
	No	97 (16.96)	25 (4.37)	127(22.2)	323(56.47)	
Demographic Factors	**Age:**					
	Underaged (<18 years)	10 (23.81)	8 (19.05)	5 (11.90)	19 (45.24)	134.99***
	Young Adult (18–35 years)	128 (5.89)	125 (5.76)	485 (22.33)	1434(66.02)	
	Middle Aged (36–55 years)	112 (5.43)	94 (4.56)	408 (19.78)	1449(70.24)	
	Older Adult (>55 years)	7(20)	13 (37.14)	5 (14.29)	10 (28.57)	
	**Gender:**					
	Male	235 (5.47)	242 (5.65)	898 (20.97)	2908 (67.89)	577.84***
	Female	12 (41.38)	8 (27.58)	5 (17.24)	4 (13.79)	
Control Variables	**Crash Time:**					
	Day	101 (5.47)	123 (6.67)	469 (25.42)	1152 (62.44)	51.57***
	Night	156 (6.23)	117 (4.74)	434 (17.59)	1760(71.34)	
	**Vehicle:**					
	CNG/Tempo	22 (3.75)	28 (4.78)	116 (19.8)	420 (71.67)	507.95***
	NMV	5 (1.8)	21 (7.55)	80 (28.78)	172 (61.87)	
	Motorcycle	3 (0.61)	25 (5.11)	146 (29.86)	315 (64.42)	
	Car/Microbus	115(21.74)	66 (12.48)	154 (29.11)	194 (36.67)	
	Medium Vehicle	45 (6.94)	41 (6.33)	126 (19.44)	436 (67.28)	
	Heavy Vehicle	67 (3.76)	59 (3.31)	281 (15.77)	1375(77.16)	
	**Collision Type:**					
	Fell off Carriageway	2 (2.47)	10 (12.35)	29 (35.80)	40 (49.38)	692.24***
	Head On	21 (6.48)	19 (5.86)	55 (16.98)	229 (70.68)	
	Hit Parked vehicle	37 (23.13)	22 (13.75)	51 (31.87)	50 (31.25)	
	Hit Pedestrian	1 (0.05)	57 (2.70)	342 (16.2)	1711 (81.05)	
	Hit Road Object	18 (24.66)	12 (16.44)	12 (16.44)	31 (42.47)	
	Overturn	4 (9.09)	5 (11.36)	6 (13.64)	29 (65.91)	
	Side Impact	10 (30.30)	7 (21.21)	8 (24.24)	8 (24.24)	
	Rear End	110 (9.86)	70 (6.27)	331 (29.66)	605 (54.21)	
	Side Swipe	54 (14.59)	38 (10.27)	69 (18.65)	209 (56.49)	
	**Traffic Control:**					
	None	47 (4.90)	49 (5.10)	181 (18.85)	683 (71.15)	240.32***
	Signage	37 (6.19)	31 (5.18)	116 (19.40)	414 (69.23)	
	Traffic Light	9 (4.39)	15 (7.32)	36 (17.56)	145 (70.73)	
	Police Controlled	103 (5.01)	113 (5.49)	493 (23.97)	1348(65.53)	
	Police and Traffic Light	33 (25.38)	7 (5.38)	14 (10.77)	76 (58.46)	
	Pedestrian Overpass	0 (0.00)	1 (2)	5 (10)	44 (88)	
	Median	8 (3.04)	19 (7.22)	54 (20.53)	182 (69.2)	
	Others (Channelization etc.)	20 (40.82)	5 (10.20)	4 (8.16)	20 (40.82)	
	**Intersection:**					
	None	100 (4.83)	92 (4.44)	410 (19.81)	1468(70.92)	72.52***
	Railway	0 (0.00)	0 (0.00)	1 (5.88)	16 (94.12)	
	Rotary	4 (2.88)	4 (2.88)	18 (12.95)	113 (81.29)	
	Staggered	6 (5.66)	5 (4.72)	21 (19.81)	74 (69.81)	
	Cross	54 (7.71)	60 (8.57)	149 (21.29)	437 (62.43)	
	Others	51 (6.92)	35 (4.75)	168 (22.8)	483 (65.54)	
	Three Leg	42 (7.73)	44 (8.10)	136 (25.05)	321 (59.12)	

Note: Values in parentheses indicate percentage of accidents based on the different levels of crash severity for various categories of each variable; ***: significant at 1% level; **: significant at 5% level; *: significant at 10% level.

About 87% of drivers involved in crashes were found not to have worn seat belts/helmets. Drivers using seat belts/helmets have lower probabilities of fatal accidents and contribute to less severe outcomes. The lack of seat belt usage relates strongly to fatal crashes. Seat belt usage has a significant effect on crash severity, as indicated by the chi-square value of 98.09 and a p-value < 0.01. The large percentage of drivers without seat belts indicates the lack of proper monitoring and traffic management rules in the city. Additionally, it is found that most of the drivers involved in the crashes did not have a valid license. This indicates the poor transport management and driver monitoring system in the context of a developing country like Bangladesh. Due to complexities in licensing and renewal, many drivers do not obtain a license, and officials in the local law enforcement agency often ignore this. Besides, many drivers in the country drive local vehicles, which are not yet defined to be licensable. This has consequences for road safety. We find that licensed drivers had lower frequencies of higher crash severities with respect to those of unlicensed drivers. About 71% of unlicensed drivers were involved in fatal crashes. Having a driver’s license is a statistically significant variable (chi-square = 558.98, p-value<0.01), showing a driver’s license as a critical factor in reducing crash severity.

Vehicle overloading by drivers is mostly done in heavy vehicles like buses and trucks. Often, bus drivers carry more passengers than the capacity to earn more ticket fares. In case of trucks, overloading of goods is done to avoid repetitive truck usage and reduce transport costs. Motorcyclists in Dhaka illegally carry more than 2 passengers in the same bike. Drivers who overloaded their vehicles were linked to fatal crashes with respect to drivers who did not overload. On the contrary, drivers without overloading their vehicles were more involved in motor collisions compared to those who overloaded. Overloading shows a strong and significant association with crash severity, with a chi-square value of 34.46 and p-value <0.01. Having a fitness certificate for vehicles seems to reduce the percentage of fatal crashes, possibly due to more cautious driving since the driver might be aware of any vehicle defect and regular maintenance. Being statistically significant, it underscored the importance of vehicle roadworthiness (chi-square = 87.91, p-value <0.01). But it can be seen that the number of drivers involved in crashes with vehicles having no fitness certificate is higher than drivers involved in crashes with vehicles of a valid fitness certificate. This also highlights the problems regarding poor transportation monitoring and law enforcement in a developing country like Bangladesh.

Overspeed was strongly associated with fatal crashes. It can be due to lower reaction times and an increase in crash impact due to high speed. A highly significant outcome by a chi-square value of 147.183 and a p-value <0.01 confirms overspeed as a critical contributor to crash severity. Drivers who overspeed often cannot judge objects in front, sideways, or the rear side of the vehicle, so they may cause major crashes. Besides, overspeeding tendencies often lead to loss of control over the vehicle, which results in crashes.

In the case of driver age, the highest frequency (50.37%) of accident occurrence has been observed in the case of young adult (18–35 years) drivers compared to the drivers of all age groups. Considering the degree of severity of the crashes, the drivers of this group exhibit that they are more prone to grievous (22.33%) and fatal (66.02%) crashes, compared to the motor collision (5.89%) and simple injury collision (5.76%), possibly due to risk-taking behavior, such as speeding and inexperience in handling unexpected or difficult situations. The lowest accident frequency has been identified in the case of older adult (above 55 years) drivers. The drivers of this group have prominent involvement in simple injury (37.14%) and fatal (28.57%) crashes, in contrast with the motor collisions (20%) and grievous injury (14.29%). The distributions of the accidents experienced by the underage drivers (below 18 years) based on the crash severity show that 45.24% of crashes are fatal, 23.81% are motor collisions, 19.05% are simple injury, and 11.90% are grievous. The middle-aged drivers (36–55 years) are significant contributors to grievous injury crashes (19.78%) and fatal crashes (70.24%), apparently due to higher exposure to traffic during work-related travel. The chi-square value of 134.99 with a p-value<0.01 indicates a significant relationship between age and crash severity. It was found that the crash frequency of male drivers was higher than that of female drivers. This is because Bangladesh does not have a driving context for women due to its socio-economic background. In many cases, females of a developing country like Bangladesh are not allowed to drive publicly on roads. The male drivers were most prone to fatal crashes, whereas female drivers had the most involvement in motor collisions. The p-value<0.01, as well as the chi-square value (577.84) indicate a highly significant association between gender and crash severity.

The control variables used in this study are accident time, vehicle type, collision type, traffic control, and intersection type. The effect of control variables is also found to be significant on crash severity from the results of the Chi-square test.

**Fig 1:**
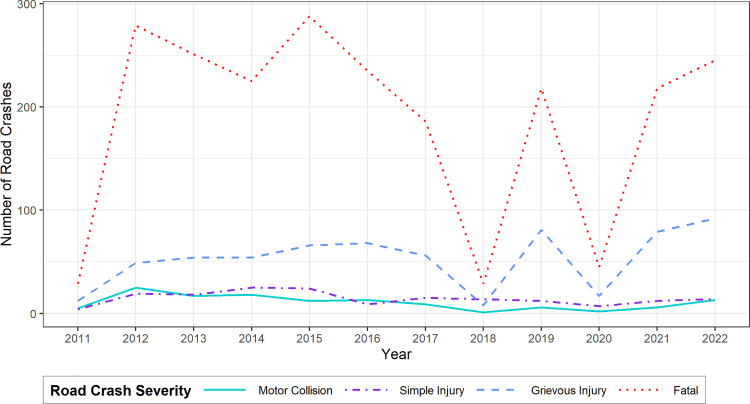
Yearly trends of road crashes of different levels of severity in Dhaka city, Bangladesh.

Fig 1 shows the yearly trends of the number of road crashes of different severities from 2011 to 2022. For all types of crash severity, we observe that road crash frequencies show increasing trends except for a massive decline in 2018 and 2020. Fatal crashes were the highest in number throughout the years, followed by grievous injury crashes. Fatal crashes decreased from the rising trend in 2014, whereas simple injuries and motor collisions increased. It can be seen that although fatal crashes increased in 2015, the frequencies started to decrease after 2015. In 2018, there was a massive student protest in Bangladesh for road safety, which spread mass awareness temporarily and was effective in reducing road crash frequencies. Therefore, fatal and grievous injury crashes went to a sharp decline in 2018. Besides, this decline can also be due to underreporting in police data, which is a limitation of many prior crash studies also [103]. The numbers again continued to increase from 2019 but declined again in 2020. Due to lockdowns imposed in response to the COVID-19 pandemic in 2020, road traffic decreased in that year. Since the movement of vehicles and pedestrians was naturally low in that year, the instances of crashes were also lower [104]. However, the number of crashes of all types of severities again started to increase from 2021.

**Fig 2:**
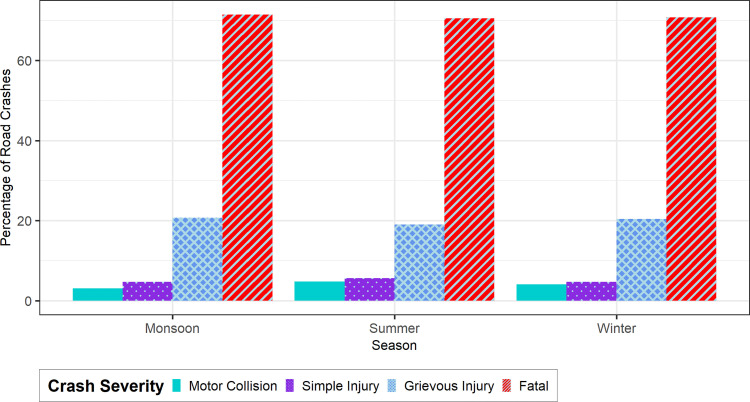
Seasonal variations of road crashes of different severities in Dhaka, Bangladesh.

Fig 2 shows the seasonal trend of road crash severities in Dhaka city. Bangladesh has broadly 3 seasonal patterns: March-June months constituting summer, July-October constituting monsoon, and November-February months constituting winter [105]. It is observed that fatal crashes are found in the highest percentage compared to other crashes of different severities across all three seasons. Fatal crashes are more in monsoon (July-October) with 71.48% of all crash severity types, followed by 70.75% in Winter (November-February) and 70.51% in Summer (March-June). Visibility problems and unfavorable weather conditions during the monsoon and winter seasons may have contributed to this. A similar pattern can be seen in the case of grievous injury crashes. Motor collision and simple injury crashes are less in number than fatal and grievous injury crashes in all seasons. The reason may be because in secondary police data, crashes of only higher severity are filed as complaint [106]. However, in summer, motor collision percentage is more than that of winter and monsoon. Summer season is generally more intense in Dhaka than the intensity of winter and monsoon, so hot weather might influence driver behavior and trigger risky driving.

**Fig 3:**
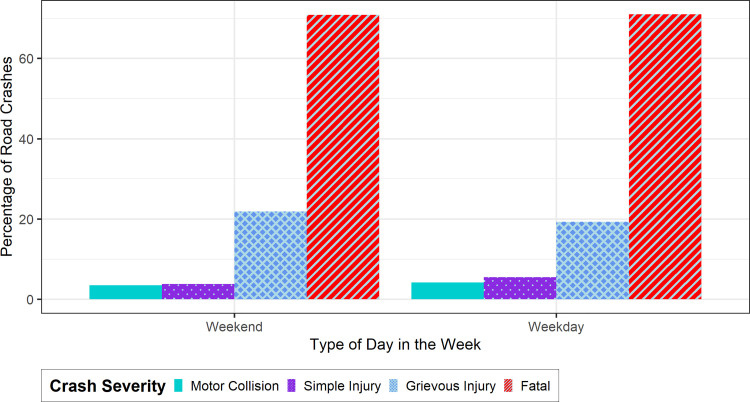
Variations of road crashes of different severities based on weekends and weekdays in Dhaka city, Bangladesh.

The weekly analysis of crash severity according to the type of day of the week has been shown in Fig 3. In Bangladesh, Fridays and Saturdays are considered as weekends and Sunday-Thursday as weekdays [107]. It was found that fatal crashes are marginally dominant on weekdays (Sunday-Thursday) in comparison to weekends (Fridays and Saturdays). Fatal crashes account for 70.82% of all crashes on weekends, and 70.95% crashes on weekdays. Grievous injury constitutes 21.83% crashes on weekdays, 19.32% crashes on weekends. The pattern of the composition of different levels of crash severity across different day types is similar to the composition across various seasons. More traffic on weekdays may lead to higher crashes. Weekend trips are generally recreational and made on low traffic conditions, and less traffic would cause fewer vehicles to collide.

**Fig 4:**
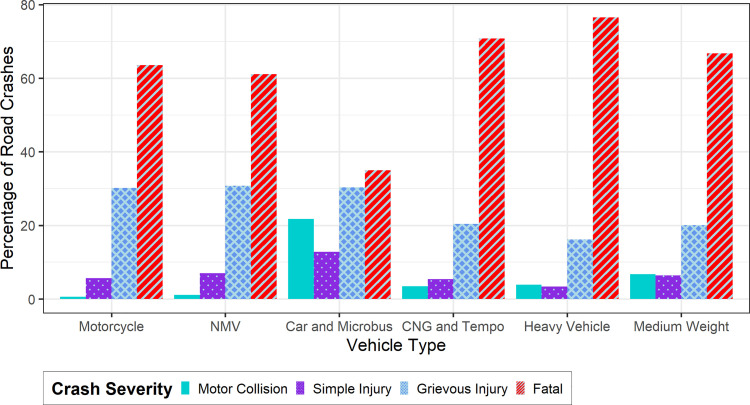
Road crash severities across vehicle types in Dhaka city, Bangladesh.

Fig 4 portrays the vehicle-specific crash severity scenario. It is observed that heavy vehicles, CNG and Tempo, and medium-weight vehicles account for a very high proportion of fatal crashes. A higher percentage of fatal crashes in heavy vehicles is because of the vehicle weight. An increase in vehicle weight may lead to more kinetic energy and momentum during a collision, which increases severity of crash. CNG and Tempo are generally light-weight vehicles, so collision of any other vehicle with CNG and Tempo may result in higher severity of crashes. Motorcycles accounted for 63.52% fatal crashes, 30.19% grievous injury crashes, 5.66% simple injury crashes, and 0.63% motor collisions of all its crash types. Motorcyclists are generally more exposed to external injuries, and they can easily overspeed. Motorcycles and NMVs have nearly the same figures. Fatal crashes account for 63.52% of crashes in motorcycles and 61.11% of crashes in NMVs. This number for grievous injury crash is 30.19% for motorcycles and 30.74% for NMVs. Cars and microbuses did not have such disproportionate distributions of crash severity types. 35.02% crashes caused by car and microbus were fatal, followed by 30.35% grievous injury, 21.79% simple injury, and 12.84% motor collisions. Cars and microbuses are of relatively stable speed and are stable stable-weight vehicles than other transport modes, so they may not have a very high difference in the composition of fatal and grievous crashes than motor collision and simple injury crashes.

### Regression results

#### Estimation of log odds and odds ratios.

The results of the ordered logit model and the generalized ordered logit model is shown in Table 4. It is found that the ordered logit model has a likelihood ratio chi-square value of 1393.69 with a p-value<0.01, indicating that the model as a whole is statistically significant. The generalized ordered logit model has a likelihood ratio Chi-square value of 1670.97, with a p-value of 0.00. Therefore, the model as a whole is statistically significant. The pseudo R-squared value for the generalized ordered logit model is 0.2121, indicating modest results. This shows that the generalized ordered logit model provides better results.

**Table 4:** Results of generalized ordered logistic regression.

Variables	Log Odds Ratio (Odds Ratio)			
	Ordered Logit Model	Generalized Ordered Logit Model		
		Motor Collision	Simple Injury	Grievous Injury
**License:** (*Reference: No)*				
Yes	−1.350*** (0.259)	−1.664*** (0.189)	−2.022*** (0.132)	−0.836*** (0.433)
**Overload:** (*Reference: No)*				
Yes	0.746*** (2.109)	−0.360 (0.697)	0.349 (1.418)	0.678*** (1.972)
**Alcohol:** (*Reference: No)*				
Yes	0.202** (1.223)	0.883*** (2.418)	0.544*** (1.722)	0.083 (1.086)
**Fitness Certificate:** (*Reference: No)*				
Yes	−0.392*** (0.675)	−0.861*** (0.423)	−0.759*** (0.468)	−0.223** (0.799)
**Seat Belt/Helmet:** (*Reference: No)*				
Yes	−0.512*** (0.598)	−0.628*** (0.534)	−0.525*** (0.591)	−0.619*** (0.538)
**Overspeed:** (*Reference: No)*				
Yes	0.651*** (1.918)	1.087*** (2.965)	0.443*** (1.558)	0.401*** (1.493)
**Age:** (*Reference: Old Age)*				
Underaged	−0.078 (0.925)	−1.514** (0.219)	−0.248 (0.779)	1.054* (2.868)
Young	0.676** (1.967)	−0.182 (0.834)	1.367*** (3.924)	1.225*** (3.404)
Middle Aged	0.646** (1.908)	−0.372 (0.689)	1.157*** (3.179)	1.26*** (3.526)
**Gender:** (*Reference: Female)*				
Male	2.285*** (9.825)	1.567*** (4.794)	2.355*** (10.539)	2.072*** (7.938)
**Light:** (*Reference: Day)*				
Night	0.482*** (1.619)	0.0434 (1.044)	0.325*** (1.384)	0.515*** (1.673)
**Vehicle:** (*Reference: CNG/Tempo)*				
NMV	0.118 (1.125)	1.536** (4.647)	0.47 (1.6)	−0.042 (0.958)
Motorcycle	0.444*** (1.559)	2.414*** (11.181)	1.026*** (2.789)	0.307* (1.359)
Car/Microbus	−1.172*** (0.309)	−1.103*** (0.332)	−1.109*** (0.329)	−1.114*** (0.328)
Medium Weight Vehicle	−0.058 (0.944)	−0.199 (0.819)	−0.043 (0.957)	−0.027 (0.974)
Heavy Weight Vehicle	0.345*** (1.412)	−0.067 (0.935)	0.364* (1.439)	0.345*** (1.412)
**Collision Type:** (*Reference: Fell off Carriageway)*				
Head On	0.637** (1.891)	−1.412* (0.244)	−0.001 (0.999)	0.936*** (2.551)
Hit Parked Vehicle	−0.896*** (0.408)	−2.305** (0.099)	−1.207*** (0.298)	−0.516* (0.597)
Hit Pedestrian	1.399*** (4.049)	1.560* (4.761)	1.488*** (4.431)	1.498*** (4.473)
Hit Road Object	−0.874*** (0.417)	−2.807*** (0.06)	−1.301*** (0.272)	0.254 (1.289)
Overturn	0.655* (1.925)	0.308 (1.361)	0.383 (1.467)	0.969** (2.636)
Side Impact (Perpendicular)	−1.054*** (0.349)	−2.985*** (0.051)	−1.648*** (0.192)	−0.527 (0.59)
Rear End	0.069 (1.071)	1.667** (0.188)	−0.026 (0.974)	0.443* (1.557)
Side Swipe	0.094 (1.098)	−2.12** (0.12)	−0.403 (0.668)	0.652** (1.92)
**Traffic Control:** (*Reference: None)*				
Signage	−0.354 (0.965)	0.555* (1.742)	0.077 (1.08)	0.003 (1.003)
Traffic Light	0.162 (1.176)	0.989* (2.688)	0.259 (1.296)	0.212 (1.237)
Police	−0.353*** (0.701)	0.078 (1.081)	−0.106 (0.899)	0.212*** (0.718)
Police and Traffic Light	−0.781*** (0.457)	−1.348*** (0.259)	−1.519*** (0.218)	−0.176 (0.838)
Pedestrian Overpass	0.848* (2.335)	11.789 (131754)	1.141 (3.128)	0.663 (1.941)
Divider	−0.102 (0.902)	0.741* (2.098)	0.065 (1.067)	−0.121 (0.886)
Others (Channelization etc.)	−1.616*** (0.198)	−0.694 (4.99)	−1.754*** (0.173)	−0.341 (0.711)
**Intersection:** (*Reference: None)*				
Railway	1.971* (7.179)	0 (1)	13.122 (500051.3)	1.895* (6.652)
Rotary	−0.507** (1.66)	0.337 (1.401)	0.017 (1.017)	0.369* (1.447)
Staggered	−0.155 (0.856)	−0.618 (0.538)	0.012 (1.012)	0.014 (1.014)
Cross	−0.343*** (0.709)	−0.177 (0.837)	−0.329** (0.719)	−0.316*** (0.728)
Others	−0.213** (0.808)	0.137 (1.145)	−0.329** (0.719)	−0.326*** (0.722)
Three Leg	−0.412*** (0.662)	−0.195 (0.823)	0.148 (1.159)	−0.109 (0.896)
Intercept	**Motor|Simple:** 0.292 **Simple|Grievous:** 1.292 **Grievous|Fatal:** 3.042	2.675** (14.508)	−1.398* (0.247)	−3.641*** (0.026)
**Model Statistics:**				
LR Chi2	1393.69	1670.97		
Prob>chi2	0.000	0.00		
Pseudo R^2^	0.1762	0.2121		

Note: Values in parentheses indicate odds ratio; ***: significant at 1% level; **: significant at 5% level; *: significant at 10% level.

First, we interpret the results of the ordered logit model. The value of the coefficient of the log odds ratio in the ordered logit model for having a license, −1.350, shows that there is a negative association between having a license and the severity of accidents. It implies that if the drivers have a license, the decrease in the log odds of experiencing a higher level of crash severity (motor collision < simple injury<grievous injury < fatal) is 1.350 compared to the drivers who do not have a license, holding all other variables constant. This decrease is statistically significant at the 1% level. The odds ratio in the ordered logit model for having a license, 0.259, indicates that, if the drivers have a license, the odds of experiencing a higher level of crash severity decrease by 74.1% ((0.259-1)*100 = −74.1%) compared to the drivers who do not have a license. This decrease is statistically significant at the 1% level. Therefore, drivers without a license have approximately 3.86 times higher odds of experiencing a more severe crash compared to licensed drivers.

The coefficient of the log-odds ratio in the ordered logit model for overload, 0.746, suggests that when the drivers overload the vehicle, the increase in the log odds of experiencing a higher level of crash severity is 0.746 compared to the drivers who do not overload, keeping other factors constant. The odds ratio for overloading, 2.109, shows that, considering all other variables the same, if the drivers overload, the odds of experiencing a higher level of crash severity increase by 110.9% ((2.109−1)*100 = 110.9%). This increase is statistically significant at the 1% level. Therefore, drivers who do not overload vehicles have approximately 0.474 times less odds of experiencing a more severe crash compared to drivers who overload.

The coefficient of the log odds ratio in the ordered logit model for alcohol shows that when the driver consumes alcohol, the increase in the log odds of experiencing a higher level of severity of accident is 0.202 compared to the drivers who do not consume alcohol, holding all other variables fixed in the model. The odds ratio for alcohol, 1.223, means that for the drivers who consume alcohol during driving, the odds of experiencing a higher category of severity of accident are 22.3%. ((1.223−1)*100 = 22.3%). This increase is statistically significant at 5% level. Therefore, drivers who do not consume alcohol have approximately 0.818 times less odds of experiencing a more severe crash compared to drivers who consume alcohol.

The coefficient in the ordered logit model for the log odds ratio for fitness certificate, −0.392 indicates that when the drivers have a valid fitness certificate, the decrease in log odds of experiencing a higher level of road crash severity is 0.392, ceteris paribus. The odds ratio for a fitness certificate, 0.675, means that if a driver has a fitness certificate for the vehicle, the odds of having a higher category of severity of accident decrease by 32.5% ((0.675-1) *100 = −32.5%). This decrease is statistically significant at 1% level, with p < 0.01. Therefore, drivers without a valid fitness certificate have approximately 1.48 times higher odds of experiencing a more severe crash than those with one.

The coefficient in the ordered logit model for the log odds ratio for seat belt, −0.512, indicates that if a driver wears a seat belt, the fall in the log odds of experiencing a higher level of crash severity is 0.512 compared to the drivers who do not wear a seat belt, holding all other variables constant. The odds ratio for seat belt of 0.598 means that if the drivers wear a seat belt, the odds of experiencing a higher category of road crash severity decrease by 40.2% ((0.598-1) *100 = −40.2%) compared to the drivers who do not wear a seat belt. Therefore, drivers who do not wear a seat belt have approximately 1.672 times higher odds of experiencing a more severe crash compared to those who wear a seat belt.

The coefficient of the log odds ratio in the ordered logit model for overspeed,0.651, indicates that when drivers overspeed, the rise in the log odds of experiencing a higher level of crash severity is 0.651compared to the drivers who do not overspeed, keeping other factors of the model constant. The odds ratio for overspeed, 1.918, means that if drivers overspeed, the odds of experiencing a higher category of severity of accident increase by 91.8% ((1.918−1) *100 = 91.8%) compared to the drivers who do not overspeed. This increase is statistically significant at 1% level. Therefore, drivers who do not overspeed have approximately 0.52 times the odds of experiencing more severe crashes than drivers who overspeed.

In the case of the variable ‘age’, old age was selected as the reference category. The coefficient in the ordered logit model of the log odds ratio for underage, −0.078, indicates that when the driver is underage (child/teenager), the change in the log odds of experiencing a higher level of severity of accident is −0.078, ceteris paribus. The odds ratio for this age group is 0.925, meaning that for underaged drivers, the odds of experiencing a higher category of crash severity decrease by 7.5% ((0.925-1)*100 = −7.5%). However, it is not statistically significant at 5% level.

The coefficient in the ordered logit model of the log odds ratio for young, 0.676, indicates that when the driver is young, the change in the log odds of experiencing a higher level of crash severity is 0.676, holding all other variables constant. The odds ratio is 1.967, meaning that for young drivers, the odds of having a higher category of severity of accident are 96.7% ((1.967− 1)*100 = 96.7%. So, the odds of having higher levels of crash severity increase by 96.7%. This increase is statistically significant at 5% level. Therefore, old drivers have approximately 0.508 times higher odds of experiencing a more severe crash compared to the young age drivers.

The coefficient of the log odds ratio in the ordered logit model for middle-aged drivers, 0.646, indicates that the change in the log odds of experiencing a higher level of severity of accident for middle-aged drivers is 0.646, when other variables are kept constant. The odds ratio of 1.908 indicates that for middle-aged drivers, the odds of having a higher level of severity increase by 90.8% ((1.908−1)*100 = 90.8%). This increase is statistically significant at 5% level. Therefore, old aged drivers have approximately 0.524 times higher odds of experiencing a more severe crash compared to middle-aged drivers.

The female gender was counted as the reference category for the variable ‘gender’. The coefficient of the log odds ratio in the ordered logit model for gender, 2.285, indicates that for male drivers, the change in the log odds of experiencing higher levels of severity of accident is 2.285, holding all other variables constant. The odds ratio for gender, 9.825, means that for male drivers, the odds of experiencing a higher category of severity of accident increase by 882.5% ((9.825−1)*100 = 882.5%). This increase is statistically significant at 1% level. Therefore, female drivers have approximately 0.102 times higher odds of experiencing a higher level of severe crash than male drivers.

We interpret the results of the generalized ordered logit model now. From the odds ratio of the generalized ordered logit model, it can be seen that drivers with a license have 0.189 odds of being in a crash severity higher than motor collision compared to unlicensed drivers. This indicates that the odds of a licensed driver to involved in a crash being a simple injury, grievous injury, or fatal decreases by 81% (1-0.189 = 0.811), holding other factors constant. Similarly, licensed drivers have 0.132 times (86.8% less) and 0.433 times (56.7% less) the odds of having a crash higher than simple injury and higher than grievous injury, respectively, than unlicensed drivers. The results for this variable were found statistically significant at 1% level of significance.

The odds ratio of overload in the generalized ordered logit model shows that drivers who overload their vehicles have 0.697 times the odds of causing a crash severity higher than a motor collision, compared with drivers who do not overload. So, the odds of simple injury, grievous injury, and fatal crash decrease by 30.3% for overloaded vehicles. This indicates that a motor collision is more likely to occur. Drivers who overload their vehicles have 1.418 times (41.8% more) the odds of crash severity higher than simple injury and 1.972 times (97.2%) the odds of crash severity higher than grievous injury. In the case of overloading, only the result under the grievous injury category was found significant at 1% level of significance, but the effects of overloading on motor collision and simple injury were found insignificant.

Drivers who consume alcohol during a crash have 2.418 times (141.8% more) the odds of experiencing a crash of a higher category than motor collision in comparison to drivers who do not consume alcohol, considering other factors constant in the generalized ordered logit model. Similarly, alcohol consumed drivers have 1.722 times the odds of experiencing a crash of higher severity than simple injury and 1.086 times the odds of experiencing a crash of higher severity than grievous injury. So, the odds of grievous injury and fatal crash increase by 72.2%, and the odds of experiencing a fatal crash increase by 8.6% for drunken drivers. However, the results of cumulative odds under grievous injury were not found to be significant, but the results for motor collision and simple injury crash were significant, with a p-value <0.01.

Drivers who drive vehicles with a valid fitness certificate have 0.423 times the odds of experiencing a crash of higher severity than a motor collision, which indicates a decrease of 57.7% in the odds of experiencing simple injury, grievous injury, and fatal crashes, holding other things constant, as found in the generalized ordered logit model. Drivers who drive vehicles with a valid fitness certificate also have 0.468 times the odds of having a crash severity higher than simple injury level and 0.799 times the odds of having a crash severity higher than grievous injury crashes, ceteris paribus. So, the odds of experiencing a grievous injury crash and a fatal crash decrease by 53.2% and the odds of experiencing a fatal crash decrease by 20.1% for drivers who drive vehicles with a valid fitness certificate when compared with drivers without a vehicle fitness. The results in all categories of crash severity were found statistically significant for this variable.

Drivers who wear a seat belt/ helmet have 0.534 times the odds of having a crash severity higher than a motor collision than drivers who do not wear a seat belt/helmet, considering other things constant in the generalized ordered logit model. So, the odds of experiencing simple injury, grievous injury, and fatal collision decrease by 46.6%. Drivers who wear a seat belt/helmet have 0.591 times (40.9% less) and 0.538 times (46.2% less) the odds of experiencing crash severity higher than simple injury and grievous injury, respectively, than drivers who do not wear a seat belt/helmet. The results for all crash severity levels were found significant at 1% level of significance for this variable.

We find from the generalized ordered logit model that drivers who overspeed vehicles have 2.965 times the odds of having a crash severity higher than a motor collision than drivers who do not overspeed, ceteris paribus. So, the odds of experiencing simple injury, grievous injury, and fatal crash increase by 196.5%. Drivers who overspeed also have 1.558 times the odds of having a crash with higher severity than simple injury and 1.493 times the odds of having a crash with higher severity than grievous injury, ceteris paribus. So, the odds of experiencing grievous injury and fatal crash increase by 55.8% and the odds of experiencing a fatal crash increase by 49.3% for drivers who overspeed. In case of overspeed, all the results were significant with p-value <0.01.

Underaged drivers have 0.219 times, 0.779 times, and 2.868 times the odds of having a crash of a higher severity level than motor collision, simple injury, and grievous injury compared to old aged drivers when other things remain constant. So, the odds of having a simple injury, grievous injury, and fatal crash decrease by 78.1%, the odds of having grievous injury and fatal crash decrease by 22.1%, but the odds of having a fatal crash increase by 186.8% for underaged drivers. The results for underaged drivers under severity levels of grievous injury crash were not statistically significant.

Young drivers have 0.834 times, 3.94 times, and 3.404 times the odds of having a crash of higher severity than motor collision, simple injury, and grievous injury when compared to old aged drivers, ceteris paribus. Therefore, young drivers have 16.6% higher odds of experiencing simple injury, grievous injury, and fatal crash, 294% higher odds of experiencing grievous injury/fatal crash and 240.4% higher odds of experiencing fatal crash than old aged drivers. The results for young-aged drivers under the motor collision category were statistically insignificant.

Middle aged drivers have 0.689 times the odds of experiencing a crash with higher severity level than motor collision, 3.179 times the odds of experiencing a crash with a higher severity level than simple injury, and 3.526 times the odds of experiencing a crash with a higher severity level than grievous injury compared to old aged drivers, holding other things constant. So, middle-aged drivers have 31.1% lower odds of having simple injury, grievous injury, and fatal crash, 217.9% higher odds of having grievous injury and fatal crash, and 252.6% higher odds of having fatal crash than old aged drivers. The results for middle-aged drivers under the simple injury category were not statistically significant.

Male drivers have 4.794 times the odds of having a crash severity higher than motor collision, 10.539 times the odds of having a crash severity higher than simple injury, and 7.938 times the odds of having a crash severity higher than grievous injury compared with female drivers, remaining other things constant in the generalized ordered logit model. So male drivers have 379.4% higher odds of having a simple injury, grievous injury, and fatal crash, 953.9% higher odds of having grievous injury and fatal crash, and 693.8% higher odds of having a fatal crash. All these results for the gender variable were statistically significant with p < 0.01.

#### Predicted probabilities.

Table 5 shows the predicted probabilities of the ordered logit model and the generalized ordered logit model. The predicted outcome for the independent variables shows the mean values for which the probabilities of different levels of crash severity was found. In the case of ordered logit mode, the margin of constant for motor collision, indicates that the probability of motor collision is 2.6% when all the predictors are set to their mean values. Similarly, the probabilities for simple injury, grievous injury, and fatal injury are 4.1%, 22.8%, and 70.4%, respectively, when all the predictor variables have mean values.

**Table 5:** Predicted probabilities of different predictors and the severity of the accident.

Variables	Predicted Outcomes			
	Motor Collision	Simple Injury Crash	Grievous Injury Crash	Fatal Crash
**License:** (*Reference: No)*				
Yes	0.148	0.148	0.148	0.148
**Overload:** (*Reference: No)*				
Yes	0.067	0.067	0.067	0.067
**Alcohol:** (*Reference: No)*				
Yes	0.188	0.188	0.188	0.188
**Fitness Certificate:** (*Reference: No)*				
Yes	0.266	0.266	0.266	0.266
**Seat Belt/Helmet:** (*Reference: No)*				
Yes	0.127	0.127	0.127	0.127
**Overspeed:** (*Reference: No)*				
Yes	0.866	0.866	0.866	0.866
**Age:** (*Reference: Old Age)*				
Underage	0.009	0.009	0.009	0.009
Young	0.504	0.504	0.504	0.504
Middle Aged	0.478	0.478	0.478	0.478
**Gender:** (*Reference: Female)*				
Male	0.993	0.993	0.993	0.993
**Light:** (*Reference: Day)*				
Night	0.569	0.569	0.569	0.569
**Vehicle:** (*Reference: CNG/Tempo)*				
NMV	0.064	0.064	0.064	0.064
Motorcycle	0.113	0.113	0.113	0.113
Car/Microbus	0.122	0.122	0.122	0.122
Medium Weight Vehicle	0.151	0.151	0.151	0.151
Heavy Weight Vehicle	0.415	0.415	0.415	0.415
**Collision Type:** (*Reference: Fell off Carriageway)*				
Head On	0.075	0.075	0.075	0.075
Hit Parked Vehicle	0.037	0.037	0.037	0.037
Hit Pedestrian	0.487	0.487	0.487	0.487
Hit Road Object	0.016	0.016	0.016	0.016
Overturn	0.01	0.01	0.01	0.01
Side Impact	0.008	0.008	0.008	0.008
Rear End	0.261	0.261	0.261	0.261
Side Impact	0.086	0.086	0.086	0.086
**Traffic Control:** (*Reference: None)*				
Signage	0.138	0.138	0.138	0.138
Traffic Light	0.046	0.046	0.046	0.046
Police	0.477	0.477	0.477	0.477
Police and Traffic Light	0.029	0.029	0.029	0.029
Pedestrian Overpass	0.012	0.012	0.012	0.012
Divider	0.061	0.061	0.061	0.061
Others	0.012	0.012	0.012	0.012
**Intersection:** (*Reference: None)*				
Railway	0.004	0.004	0.004	0.004
Rotary	0.033	0.033	0.033	0.033
Staggered	0.025	0.025	0.025	0.025
Cross	0.162	0.162	0.162	0.162
Others	0.171	0.171	0.171	0.171
Three Leg	0.127	0.127	0.127	0.127
**Ordered Logit Model Constant:**				
Margin	0.026***	0.041***	0.228***	0.704***
Std. Error	0.002	0.003	0.007	0.008
**Generalized Ordered Logit Model Constant:**				
Margin	0.008	0.040	0.254	0.698***
Std. Error	0.067	0.205	0.194	0.008

Note: ***: significant at 1% level; **: significant at 5% level; *: significant at 10% level.

In case of generalized ordered logit model, the margin of constant for motor collision, simple injury crash and grievous injury crash shows that their corresponding probabilities are 0.8%, 4%, 25.4% respectively. However, these values are not significant in the case of the generalized ordered logit model. In case of fatal crash, the predicted probability is 69.8%, which is significant at 1% level of significance.

#### Marginal effects.

Table 6 shows the marginal effects of the ordered logit model. The marginal effects of the ordered logit model suggest that drivers who have a license are 28.2 percentage points less likely to cause a fatal injury collision, 19.5 percentage points more likely to cause a grievous injury collision, 5.13 percentage points more likely to cause simple injury collision, and 3.4 percentage points more likely to cause a motor collision than non-licensed drivers. The effects are statistically significant at 1% level.

**Table 6:** Marginal effects of predictor variables on accident severity for the ordered logit model.

Variables	dy/dx			
	Motor Collision	Simple Injury Crash	Grievous Injury Crash	Fatal Crash
**License** (*Reference: No)*				
Yes	0.034***	0.0513***	0.195***	−0.282***
**Overload** (*Reference: No)*				
Yes	−0.019***	−0.028***	−0.108***	0.156***
**Alcohol** (*Reference: No)*				
Yes	−0.005**	−0.008**	−0.029**	0.042**
**Fitness Certificate:** (*Reference: No)*				
Yes	0.010***	0.015***	0.056***	−0.082***
**Seat Belt/Helmet:** (*Reference: No)*				
Yes	0.013***	0.019***	0.074***	−0.107***
**Overspeed:** (*Reference: No)*				
Yes	−0.017***	−0.025***	−0.094***	0.135***
**Age:** (*Reference: Old Age)*				
Underage	0.002	0.003	0.011	−0.016
Young	−0.017**	−0.026**	−0.098**	0.140*
Middle Aged	−0.016**	−0.025**	−0.093**	0.134**
**Gender:** (*Reference: Female)*				
Male	−0.058***	−0.087***	−0.331***	0.476***
**Light:** (*Reference: Day)*				
Night	−0.012***	−0.018***	−0.069***	0.101***
**Vehicle:** (*Reference: CNG/Tempo)*				
NMV	−0.003	−0.004	−0.017	0.025
Motorcycle	−0.011***	−0.017**	−0.065***	0.092***
Car/Microbus	0.029***	0.044***	0.17***	−0.244***
Medium Weight Vehicle	0.001	0.002	0.008	−0.012
Heavy Weight Vehicle	−0.009***	−0.131***	−0.05***	0.072***
**Collision Type:** (*Reference: Fell off Carriageway)*				
Head On	−0.016**	−0.024**	−0.092**	0.132**
Hit Parked Vehicle	0.023***	0.034***	0.13***	−0.186***
Hit Pedestrian	−0.036***	−0.531***	−0.202***	0.291***
Hit Road Object	0.022***	0.033***	0.127***	−0.182***
Overturn	−0.017*	−0.025*	−0.095*	0.136*
Side Impact	0.0269***	0.040***	0.153***	−0.219***
Rear End	−0.001	−0.003	−0.01	0.014
Side Swipe	−0.002	−0.004	−0.013	0.019
**Traffic Control:** (*Reference: No Traffic Control)*				
Signage	0.009	0.001	0.005	−0.007
Traffic Light	−0.004	−0.006	−0.023	0.034
Police	0.009***	0.013***	0.051***	−0.074***
Police and Traffic Light	0.019***	0.029***	0.113***	−0.162***
Pedestrian Overpass	−0.022*	−0.032***	−0.122*	0.177*
Divider	0.003	0.004	0.014	−0.021
Others (Channelization etc.)	0.041***	0.061***	0.235***	−0.337***
**Intersection:** (*Reference: No Intersection)*				
Railway	−0.050*	−0.749*	−0.285*	0.411*
Rotary	−0.129**	−0.019**	−0.074**	0.105**
Staggered	0.004	0.006	0.0225	−0.032
Cross	0.009***	0.130***	0.0498***	−0.071***
Three Leg	0.010***	0.016***	0.059***	−0.086***
Others	0.005**	0.008**	0.031**	−0.045**

Note: ***: significant at 1% level; **: significant at 5% level; *: significant at 10% level.

The marginal effects of the ordered logit model shows that drivers who overload their vehicles have 15.6 percentage points more likelihood of a fatal collision than drivers who do not overload. On the other hand, overloaded drivers are 1.9, 2.8, and 10.8 percentage points less likely to be involved in the incidence of motor collision, simple injury collision, and grievous injury collision, respectively, than the drivers who do not overload. The effects are statistically significant at 1% level. This indicates that drivers of overloaded vehicles are more likely to cause fatal accidents than those of lower-severity crashes, which may be due to the slower speeds of overloaded vehicles.

Drivers who consume alcohol during an accident are 4.2 percentage points more likely to cause fatal accidents than drivers who do not consume alcohol. The coefficient is statistically significant at a 5% level. Drunk driving is hence a big cause of fatal RTAs. We find the opposite directions for the marginal effects of the ordered logit model in the case of other severity levels of alcohol consumed drivers.

Drivers who have a fitness certificate are 8.2 percentage points less likely to cause a fatal injury collision. Drivers who wear seat belts (or helmets for motorcyclists) have 10.7 percentage points less likelihood of a fatal injury collision than the drivers who do not wear seat belts, and the effect is significant at 1% level. Overspeeding behavior of drivers poses 13.5 percentage points more likelihood of fatal injury collision than the drivers who do not overspeed, and the effect is significant at 1% level.

The marginal effects of the ordered logit model show that underaged drivers have 0.2 percentage points more likelihood of motor collision, 0.3 percentage points more likelihood of simple injury collision, 1.1 percentage points more likelihood of grievous injury collision, and 1.6 percentage points less likelihood of fatal injury collision than older drivers. Therefore, underage drivers are less likely to be involved in accidents of a higher level of crash severity. The effects are statistically significant at the 10% level. But young drivers have 14 percentage points more likelihood of fatal injury accident, 9.8 percentage points less likelihood of grievous injury accident, 2.6 percentage points less likelihood of simple injury accident, and 1.7 percentage points less likelihood of motor collision than older drivers. The effects are statistically significant at the 10% level for fatal collision and the 5% level of significant for other degrees of severity. So, young drivers have a higher risk of engaging in fatal and serious injury accidents than lower-severity crashes. The middle-aged drivers have 1.6 percentage points less likelihood of motor collision, 2.5 percentage points less likelihood of a simple injury accident, 9.3 percentage points less likelihood of a grievous injury accident, but 13.4 percentage points more likelihood of fatal accidents than older drivers. The effects are statistically significant at 5% level.

Male drivers are 47.6 percentage points more likely to indulge in a fatal collision but 5.8 percentage points less likely to indulge in a motor collision, 8.7 percentage points less likely to indulge in a simple injury collision, and 33.1 percentage points less likely to indulge in a grievous injury collision than female drivers.

The marginal effects of the generalized ordered logit model are shown in Table 7. The marginal effects of the generalized ordered logit model show that drivers who have a license are 1.3 percentage points more likely to have a motor collision, 8 percentage points more likely to have a simple injury crash and 8.3 percentage points more likely to have grievous injury crash than drivers without a license. However, these are not statistically significant. But drivers with a license are 17.6 percentage points less likely to involve in fatal crash than drivers without a license, which is statistically significant at 1% level of significance.

**Table 7:** Marginal effects of predictor variables on accident severity for the generalized ordered logit model.

Variables	dy/dx			
	Motor Collision	Simple Injury	Grievous Injury	Fatal Crash
**License** (*Reference: No)*				
Yes	0.013	0.080	0.083	−0.176***
**Overload** (*Reference: No)*				
Yes	0.0029	−0.0190	−0.1270*	0.1432***
**Alcohol** (*Reference: No)*				
Yes	−0.007	−0.018	0.008	0.017
**Fitness Certificate:** (*Reference: No)*				
Yes	0.0069	0.0281	0.0120	−0.0471**
**Seat Belt/Helmet:** (*Reference: No)*				
Yes	0.005	0.019	0.106	−0.131***
**Overspeed:** (*Reference: No)*				
Yes	−0.0087	−0.0117	−0.0641	0.0845***
**Age:** (*Reference: Old Age)*				
Underage	0.0121	−0.0007	−0.2336**	0.222*
Young	0.0014	−0.064	−0.195	0.258***
Middle Aged	0.0029	−0.0563	−0.2124	0.266***
**Gender:** (*Reference: Female)* Male	−0.0126	−0.0961	−0.3282	0.4368***
**Light:** (*Reference: Day)* Night	−0.0003	−0.0147	−0.0936*	0.1086***
**Vehicle:** (*Reference: CNG/Tempo)*				
NMV	−0.012	−0.009	0.031	−0.009
Motorcycle	−0.019	−0.028	−0.017	0.065*
Car/Microbus	0.009	0.042	0.184	−0.235***
Medium Weight Vehicle	0.0016	0.0004	0.0036	−0.0056
Heavy Weight Vehicle	0.001	−0.017	−0.056	0.073***
**Collision Type:** (*Reference: Fell off Carriageway)*				
Head On	0.0113	−0.0113	−0.1975***	0.1975***
Hit Parked Vehicle	0.0185	0.0372	0.0531	−0.1088*
Hit Pedestrian	−0.0125	−0.0562	−0.2472	0.3158***
Hit Road Object	0.0225	0.0375	−0.1136	0.0536
Overturn	−0.0025	−0.0152	−0.1867*	0.2044**
Side Impact	0.0239	0.0521	0.0352	−0.1112
Rear End	0.0134	−0.0121	−0.0946**	0.0934*
Side Swipe	0.0170	0.0016	−0.1562*	0.1376**
**Traffic Control:** (*Reference: No Traffic Control)*				
Signage	−0.004	0.001	0.003	0.001
Traffic Light	−0.008	−0.004	−0.033	0.045
Police	−0.001	0.006	0.065**	−0.070***
Police and Traffic Light Pedestrian Overpass	0.011 −0.094	0.059 0.042	−0.033 −0.087	−0.037 0.140
Divider	−0.006	0.003	0.029	−0.026
Others (Channelization etc.)	0.006	0.075	−0.009	−0.072
**Intersection:** (*Reference: No Intersection)*				
Railway	0.000	−0.605	0.206	0.400*
Rotary	−0.003	0.002	−0.077*	0.078*
Staggered	0.005	−0.006	−0.002	0.003
Cross	0.001	0.014	0.052	−0.067***
Three Leg	0.002	−0.008	0.030	−0.023
Others	−0.001	0.016	0.053	−0.069***

Note: ***: significant at 1% level; **: significant at 5% level; *: significant at 10% level.

Drivers who overload their vehicles are 0.29 percentage points more likely to involve in motor collision and 1.9 percentage points less likely to involve in simple injury collision compared to drivers who do not overload vehicles, but these results are not statistically significant. But drivers who overload are 12.7 percentage points less likely to cause a grievous injury crash and 14.32 percentage more likely to cause a fatal crash, which are statistically significant in the generalized ordered logit model.

Drivers who consume alcohol at the time of crash are 0.7 percentage points less likely to involve in motor collision, 1.8 percentage points less likely to involve in simple injury crash, 0.8 percentage points more likely to involve in grievous injury crash and 1.7 percentage points more likely than drivers who do not consume alcohol during driving to involve in fatal crash. But none of the results from alcohol consumption was found significant in the marginal effects of the generalized ordered logit model.

Drivers with a fitness certificate are 0.69 percentage points more likely to have motor collision, 2.81 percentage points more likely to have simple injury crash, 1.2 percentage points more likely to involve in grievous injury crash than drivers without fitness certificate. However, these results are not statistically significant in the marginal effects of generalized ordered logit model. But in case of fatal crash, significant results were found, which showed that drivers with fitness certificate are 4.71 percentage points less likely to indulge in a fatal crash than drivers without a fitness certificate.

Drivers who wear seat belt/helmet during the time of crash are 5 percentage points more likely to involve in a motor collision, 1.9 percentage points more likely to involve in a simple injury collision and 10.6 percentage points more likely to involve in grievous injury crash than drivers who do not wear seat belt/helmet. Drivers who wear seat belt/helmet are 13.1 percentage points less likely to involve in fatal crash than drivers who do not wear seat belt/helmet.

In case of over speeding behavior, it can be seen from the marginal effects of the generalized ordered logit model that drivers who overspeed their vehicles are 8.45 percentage points more likely to involve in a fatal crash than drivers who do not overspeed their vehicles. However, over speeding drivers may be 0.87 percentage points less likely to involve in motor collision, 1.17 percentage points less likely to involve in simple injury crash, 6.41 percentage points less likely to involve in grievous injury crash than drivers who do not overspeed.

Underaged drivers are 1.21 percentage points more likely than old aged drivers to involve in a motor collision, 0.07 percentage points less likely to involve in simple injury crashes. Underaged drivers are also 23.36 percentage points less likely to involve in grievous injury crashes and 22.2 percentage points more likely to indulge in fatal crashes than old aged drivers and these findings are statistically significant. Young drivers are 0.14 percentage points more likely to involve in motor collision, 6.4 percentage points less likely to involve in simple injury crash and 19.5 percentage points less likely to involve in grievous injury crash than olds aged drivers. Young drivers are 25. 8 percentage points more likely to involve in fatal crashes than old aged drivers. Middle aged drivers are 0.29 percentage points more likely to involve in motor collision, 5.63 percentage points less likely to involve in simple injury crashes and 21.24 percentage points less likely to involve in grievous injury crashes than old aged drivers. Middle aged drivers are 26.6 percentage points more likely to cause a fatal crash than old aged drivers, which was found statistically significant at 1% level of significance.

Male drivers are 1.26 percentage points less likely than female drivers to involve in motor collision, 9.61 percentage points less likely to involve in simple injury crash and 32.82 percentage points less likely to involve in grievous injury crash. Male drivers are 43.68 percentage points more likely than female drivers to cause a fatal crash, which is significant at 1% level of significance in the case of the generalized ordered logit model.

## Discussion

### Discussion on results

The findings of this study emphasize the impact of behavioral and demographic factors on road crash severity, offering new insights into the dynamics of crash outcomes. This study answers the question of what significant factors cause a driver to be involved in higher levels of crash severity in an urban setting. We explain the findings of the ordered logit model and generalized ordered logit model in this section.

The findings show that licensed drivers are associated with a decrease in the likelihood of higher levels of accident severity, both in the ordered logit and generalized ordered logit model. This is because licensed drivers are trained in safe driving practices, whereas unlicensed drivers may not have the correct driving skills and may also be unaware of road environments. This is supported by Hanna et al. [108], who revealed that unlicensed drivers are more likely to engage in unsafe driving behaviors, leading to an increased probability of severe traffic accidents. In a study conducted by Bhuiyan et al. [18] in Bangladesh, it was also found that professional drivers with valid licenses are less likely to be involved in severe crashes. The presence of unlicensed drivers on the road indicates a lack of supervision and regulation regarding who can drive on urban roads. Therefore, traffic injury prevention programs should aim to bring all drivers under a valid licensing scheme through examination and training.

Drivers who overload vehicles increase the odds of higher level of crash severity, both in the case of ordered logit and generalized ordered logit model. This aligns with the higher risk associated with overloaded vehicles stemming from maneuvering difficulties and increased stopping distances. This is supported by Zhang et al. [84], who discovered that overloaded vehicles are involved in more severe crashes compared to cases of drunk driving. This could be attributed to vehicle malfunctions and challenges in control and braking resulting from overloading. Overloading is predominantly observed in freight vehicles and public transport. For example, buses carry more passengers than the permitted seat occupancy and standing capacity allow, aiming to generate more ticket revenues. These passengers often carry bulky bags and materials, adding pressure to the vehicle’s smooth movement. Drivers should face penalties for overloading vehicles to prevent crashes caused by excessive loads.

It was found that during the study period, the total number of drivers who had consumed alcohol was lower than non-consumers. However, even though they were a small group, alcohol-impaired drivers showed a higher likelihood of being involved in higher-level severe accidents. This emphasizes the negative impact of alcohol consumption on reaction times and judgment. This conclusion is supported by Pervaz et al. [17] and Hingson and Winter [109], who also found that alcohol- impaired drivers are more likely to be in severe accidents. These results contrast with the surprising findings of Lee et al. [56], who suggested that drunk drivers drive slowly and are involved in less severe accidents. To address this issue, traffic and highway police could be tasked with stopping and penalizing vehicles suspected of being driven by alcohol-impaired drivers.

Drivers who drive and maintain vehicles with a valid fitness certificate show a reduced likelihood of a higher level of crash severity. It is confirmed by both the ordered logit and the generalized ordered logit model. This is because drivers who hold a fitness certificate are aware of their vehicles and are less likely to be involved in crashes that cause damage to their vehicles. This result is aligned with Pervaz et al. [17], who suggest that vehicle defects have been identified as contributing factors to crash severity, implying that the lack of a fitness certificate could be associated with higher risks. Defective vehicles may not run fully under the control of the driver. It is also the responsibility of the driver to test whether their vehicle is fully functional before starting the vehicle. Not having a fitness certificate implies that the driver is operating a vehicle that she/he know is not fully functional. It is a major driver error to operate a vehicle with known defects. Inspection stations by transport police should examine vehicles that appear to have faulty movements.

Not using seat belts (or helmets for motorcyclists) is associated with significantly higher odds of a higher degree of accident severity. This underscores the critical role of seat belts in mitigating injury severity during crashes, as found by Febres et al. [110], who concluded that failure to wear a seat belt significantly increases the risk of fatal and severe injury in traffic accidents. Not using a seat belt may not be completely due to the driver’s fault. If the vehicle lacks a seat belt or has a malfunctioning seat belt mechanism, then severe injuries may occur. This is substantiated by Klinjun et al. [52], who stated that malfunctioning seat belts may also cause crashes. Therefore, traffic police should check whether the driver is wearing a seatbelt on the road. Additionally, all motorcyclists without helmets have to be identified and penalized to prevent severe crashes. Law enforcement inspections can also check whether vehicles on the road have functioning seat belts.

The odds ratio of overspeed in both ordered logit and generalized ordered logit model implies that the overspeeding behavior of the driver increases the likelihood of a higher degree of accident severity. This finding is supported by Dabbour et al. [78] and Ma et al. [65]. High speeds reduce reaction times and increase crash impact forces, making speed management a key area for intervention. Ferguson [111] also found that speeding is identified as a major risk factor, especially for young drivers, contributing to higher crash risk and severity. Moreover, Adanu et al. [53] stated that overspeeding increases the risk of a higher severity of accident by 2.17 times. Often, overspeeding may be caused by other factors, such as alcohol consumption, the age of the driver, vehicle defects, etc.

The results show that young adults are significantly more likely to be involved in severe accidents compared to children and older adults. The records of young drivers’ involvement in road crashes are also higher than other age groups in the study period. Young adults often exhibit risk-taking behavior. These findings align with Ferguson [111], who found that young drivers, especially those newly licensed, have a very high crash risk, with age being a significant factor in accident severity. Moreover, Borhan et al. [77] state that young people have naturally more risk-taking tendencies, possibly due to their lack of understanding of the consequences of their actions. However, the findings of our study contrast with those of Classen et al. [112], who suggest that older adults may also show higher crash severity due to factors like diminished cognitive abilities. Dabbour et al. [78] stated that both young and old-aged drivers contribute to severe crashes, but in our study, it was found that old-aged drivers have less frequency of involvement in accident cases in the study period, so elderly drivers are less likely to be involved in severe crashes. Middle-aged drivers are also found to have a positive association with higher levels of severity of crashes in our study. From marginal effect analysis, it was found that young-aged and middle-aged drivers have a higher likelihood of fatal injury accidents than older-aged drivers. So, it can be inferred that both young and middle-aged drivers are responsible for higher levels of severity of RTAs in Dhaka.

Male drivers are more likely to be involved in severe accidents than female drivers, reflecting higher instances of risky driving behaviors among men. This is because male drivers often engage in risky driving practices such as aggressive driving and overspeeding tendencies [35]. The findings are aligned with research contributed by Cespedes et al. [39] and Lardelli-Claret et al. [79]. However, while females are less likely to cause accidents, they may be more vulnerable as victims of road crashes. Bose et al. [113] discovered that female drivers face a greater risk of injury or fatality in accidents. Therefore, initiatives aimed at preventing road traffic accidents should focus on curbing risky behavior among male drivers and ensuring the safety of female drivers.

The control variables exhibit valuable inferences. Driving at night is associated with a higher likelihood of crash severity. Reduced visibility and driver fatigue can be contributing factors. Moreover, Plainis et al. [114] found that driving at night is associated with a higher likelihood of severe crashes and that a disproportionate number of fatal injuries occur after dark. However, Adeyemi et al. [115] suggest that driving during the morning rush hour is also associated with an increased likelihood of severe accidents due to aggressive behavior. But from our study, it can be confirmed that nighttime crashes are more severe than daytime crashes. The reason may be driver fatigue or visibility problems at night. Klinjun et al. [52] found that longer driving durations may induce drowsiness and fatigue in drivers, which may lead to distracted driving. Drivers of heavy vehicles are more likely to be involved in severe crashes, likely due to vehicle size and momentum. This complies with Safari et al. [116], who indicate that vehicle characteristics can significantly affect crash severity, with heavier vehicles often associated with more severe outcomes. However, Roudsari et al. [117] found that lighter vehicles are more prone to severe accidents, particularly in collisions with larger vehicles. Lighter vehicles like motorcycles exhibit differing risks, emphasizing the need for targeted safety measures. In addition, Ahmed et al. [30] suggest that NMVs, such as bicycles and animal-drawn carts, are more prone to severe accidents compared to motorized vehicles. Thus, the effect of vehicle characteristics on driver faults may differ with contexts. Head-on collisions and pedestrian impacts are the most severe type of statistically significant crash types. These findings highlight the importance of mitigating high-impact collision scenarios. The finding complies with Mussone et al. [118], where it is found that certain collision types, such as head-on collisions, are associated with higher injury severity. Reith et al. [119] concluded that pedestrians involved in vehicle collisions often experience higher injury severity and mortality rates compared to occupants in vehicle-to-vehicle crashes. Head-on crashes can be due to a big fault of drivers, since this type of collision occurs due to non-compliance with road traffic regulations. Drivers who hit pedestrians may do so because of poor visibility of their surroundings or due to aggressive driving. Intersections without adequate traffic control measures (e.g., signage or signals) show odds of higher accident severity. The presence of police or pedestrian overpasses moderately mitigates crash outcomes. This complies with Haleem and Abdel-Aty [120], who suggest that unsignalized intersections often experience higher crash severity due to the lack of regulated vehicle movements, thus highlighting the elevated risks in such environments. Pedestrian overpass as a traffic safety technique is also found in our study to significantly reduce RTA severity.

### Policy implications

The findings of this paper shed light on how different driver characteristics contribute to crash severity. The outcomes of the study can help to design effective RTA prevention schemes and formulate policies. This research work can help transport planners, engineers, accident researchers, and policy-makers in their work regarding accident analysis. From our study, we found that alcohol consumption and not using seatbelts increase the severity of crashes. In Brazil, a zero-tolerance drinking and driving law was effective in Brazil to reduce road crash severity [121]. In Dhaka, alcohol consumption act and law can be formulated to check driving under influence. At workplaces, drivers can undergo regular testing to identify addiction and drug abuse. Alcohol and drug screening helps to reduce crash severity [122]. Besides, it is possible to conduct road-side drug tests [123], which can be done by law enforcement agencies on doubt. In case of seat belt use, strong enforcement of law and use of task force to ensure seat belt use can be adopted [124]. Besides, visual and audio based warning and notification can be provided at streets to drivers. In case of motorcycle drivers, mandatory helmet use laws and enforcement can be ensured. Research has shown that mandatory helmet use schemes are useful in reducing crash severities [125]. In Bangladesh, currently regulatory policies have started ‘no helmet, no fuel’ strategy for motorcyclists [126]. This mandate can be extended to ‘no helmet, no ride’ strategies, where no motorcyclist without helmet will be allowed to use roads. In case of young drivers, their behavior needs to be controlled through some secondary intervention schemes. In this case, education and training of young drivers can be useful. No young aged drivers can be allowed to drive without a certified training on road safety. In addition, technological strength can be useful. For example, Camden et al. [127] found that web based instruction programs for young drivers can reduce their risky behaviors on roads. In case of fitness certificate of vehicles and licensing, the road vehicle and transportation concerned authorities can initiate monitoring and examination programs to identify unfit vehicles and drivers without license. Unlicensed drivers can be given monetary charges, and trained to achieve legal license. Vehicle overloading can be monitored using advanced intelligent systems. Intelligent detector technology such as microcontroller-based systems can be helpful in this regard [128]. In this case, police and transportation authority can set up monitoring sensors at specified road points to identify overloaded vehicles and stop those vehicles. National policies can aim to utilize these strategies.

From a general perspective, we recommend routine vehicle inspections to ensure fitness certification, license check, as well as technology-driven solutions like speed cameras, adaptive traffic signals, and advanced driver-assistance systems can effectively monitor and control unsafe driving. Most notably, improved driver training emphasizing defensive driving, adherence to traffic laws, and hazard recognition, particularly for young and inexperienced drivers, may be effective. Public awareness campaigns to enlighten people about seatbelt usage, the dangers of speeding, and the risks of drunk driving can foster a culture of safety among all road users and cause voluntary involvement in safe practices. Above all, we suggest the preparation of an urban road safety strategic plan for Dhaka city, which will clearly outline the road safety design and regulatory schemes to reduce the instances of road crashes and related social adversities.

## Conclusion

This study aimed to evaluate the behavioral and demographic determinants of road crash severity using the crash data of the DMP recorded from 2011 to 2022 in Dhaka city, Bangladesh. In this study, we used ordered logistic regression and generalized ordered logistic regression as econometric models of analysis, which were interpreted using log-odds and odds ratios, predicted probabilities, and marginal effects. We identified two demographic (age, gender) and six behavioral (alcohol consumption, seatbelt/helmet use, overloading, overspeeding, fitness certificate, licensing) determinants of road crash severity.

We found that young and middle-aged drivers show a higher risk of severe accidents compared to other age groups. This is likely due to the risk-taking nature of younger drivers and increased road exposure for the middle-aged due to their driving requirements in daily life. Additionally, male drivers are more prone to severe crashes than females, indicating a greater tendency towards risky behaviors and aggressive driving tendencies by males. Behavioral factors, such as overloading vehicles and not using seatbelts, also contribute significantly to crash severity. Alcohol consumption and overspeeding were highly responsible for the higher severity of crashes. We also found that drivers without a valid license and driving vehicles without proper fitness certification are associated with higher severities. These findings highlight the importance of considering human behavior, driving conditions, and inadequacies of technology and infrastructure in determining crash outcomes.

Addressing these challenges requires a comprehensive approach involving targeted policy measures, infrastructure development, and behavioral interventions. Strict enforcement of traffic laws is crucial, especially concerning behaviors like speeding, overloading, and drunk driving. In addition, we recommend the preparation of a road safety strategic plan, including age and gender specific guidelines. New and upcoming technologies can be incorporated with regulatory and design schemes to monitor risky drivers and identify risky places, which will be prioritized for instantaneous safety interventions.

Because of data unavailability, the study was confined to the capital city, Dhaka, only. Future studies could extend to other regions of the country, subject to data availability. Due to a lack of rigorous crash reporting and insufficient monitoring, important variables like driver income, household characteristics, marital status, residential status, years of experience, and education on driving rules were not included in this study. These are the limitations in the context of a developing country like Bangladesh, where data availability becomes a big challenge. Future research can use natural language processing and a large language model to extract additional information from various sources.The instantaneous factors for road crash severity could be studied by utilizing vision language model from live CCTV footage and surrogate safety measures, or a primary survey in the future. Additionally, emerging crash-prone vehicles like auto-rickshaws, where drivers lack formal licensing and tend to overspeed due to vehicle characteristics, seasonal variations of driving behavior, were not analyzed. Future researchers can build on these limitations to conduct further studies on evolving factors of crash severity.

## Supporting information

S1 Data DMP-2011. (XLSX)

S2 Data DMP-2012. (XLSX)

S3 Data DMP-2013. (XLSX)

S4 Data DMP-2014. (XLSX)

S5 Data DMP-2015. (XLSX)

S6 Data DMP-2016. (XLSX)

S7 Data DMP-2017. (XLSX)

S8 Data DMP-2018. (XLSX)

S9 Data DMP-2019. (XLSX)

S10 Data DMP-2020. (XLSX)

S11 Data DMP-2021. (XLSX)

S12 Data DMP-2022. (XLSX)
